# Curdlan Limits *Mycobacterium tuberculosis* Survival Through STAT-1 Regulated Nitric Oxide Production

**DOI:** 10.3389/fmicb.2019.01173

**Published:** 2019-05-28

**Authors:** Shikha Negi, Susanta Pahari, Deepjyoti Kumar Das, Nargis Khan, Javed N. Agrewala

**Affiliations:** ^1^Immunology Division, CSIR - Institute of Microbial Technology, Chandigarh, India; ^2^Immunology Division, Texas Biomedical Research Institute, San Antonio, TX, United States; ^3^Department of Microbiology and Immunology, McGill University, Montreal, QC, Canada; ^4^Department of Biomedical Engineering, Indian Institute of Technology Ropar, Rupnagar, India

**Keywords:** macrophages, curdlan, iNOS, T cells, host-directed therapy, tuberculosis

## Abstract

Host-directed therapies have emerged as an innovative and promising approach in tuberculosis (TB) treatment due to the observed limitations of current TB regimen such as lengthy duration and emergence of drug resistance. Thus, we explored the role of curdlan (beta glucan polysaccharide) as a novel strategy to activate macrophages against *Mycobacterium tuberculosis* (*Mtb*). The aim of the study was to investigate the role of curdlan in restricting the *Mtb* growth both *in vitro* and *in vivo*. Further, the immunomodulatory potential of curdlan against *Mtb* and the underlying mechanism is largely unknown. We found that curdlan treatment enhanced the antigen presentation, pro-inflammatory cytokines, *Mtb* uptake and killing activity of macrophages. *In vivo* studies showed that curdlan therapy significantly reduced the *Mtb* burden in lung and spleen of mice. Administration of curdlan triggered the protective Th1 and Th17 immunity while boosting the central and effector memory response in *Mtb* infected mice. Curdlan mediated anti-*Mtb* activity is through signal transducer and activator of transcription-1 (STAT-1), which regulates nitric oxide (NO) production through inducible NO synthase (iNOS) induction; along with this activation of nuclear factor kappa B (NF-κB) was also evident in *Mtb* infected macrophages. Thus, we demonstrate that curdlan exerts effective anti-tuberculous activity anti-tuberculous activity. It can be used as a potential host-directed therapy against *Mtb*.

## Introduction

Tuberculosis (TB) continues to be the devastating infectious disease with the highest mortality and morbidity after HIV (Glaziou et al., 2018). Owing to its poor diagnosis and long regimen of drug treatment, resistant strains of *Mycobacterium tuberculosis* (*Mtb*) are likely to develop in the host (Gandhi et al., 2010; Lawn and Zumla, 2011; Prabowo et al., 2013). Among *Mtb* infected individuals, only 10–15% develops the disease during their lifetime, rest remains protected until the resurgence in their immunity (Alene et al., 2018). Thus, there is a need to understand the intricate interaction between pathogen and host immune factors, which regulates the disease pathogenesis and outcome. This would aid to design novel host-directed therapeutic interventions to control TB. An effective approach is to stimulate the host immune system against *Mtb*.

Macrophages (MΦs) are the key players of immune system but also a favorable intracellular niche for *Mtb* (Pieters, 2008; Pahari et al., 2018). They express array of PRRs such as toll-like receptor (TLR-2, 3, 4, 5, 7, 8, and 9), nucleotide-binding oligomerization domain (NOD-1 and NOD-2) protein like receptors and C- type lectin receptors (CLRs; mincle, dectin-1, dectin-2) (Pasare and Medzhitov, 2004; Kumar et al., 2013). PRRs on immune cells recognize conserved patterns such as pathogen-associated molecular patterns (PAMPs) or synthetic ligands, which triggers their activation against *Mtb* (Pahari et al., 2017). Host-directed therapies employing immunomodulators that targets PRRs can be an effective strategy to control *Mtb* burden and emergence of drug-resistant strains of *Mtb*.

Beta glucans are known to bind PRRs and elicit host defense responses (Vannucci et al., 2013). Curdlan is a high molecular weight beta 1–3 glucan polysaccharide, extracted from non-pathogenic strains of the bacterium *Alcaligenes faecalis* (Zhang and Edgar, 2014). It has been approved by FDA owing to its safety as food additive and low cost of production (Spicer et al., 1999). It is known to stimulate dectin-1, a C-type lectin receptor that signals through Syk (Xie, 2012). Dectin-1 is expressed on myeloid cells including MΦs and DCs (Taylor et al., 2002). *Mtb* interaction with dectin-1 has been implicated in induction of Th1/Th17 immunity and higher production of IL-12 cytokine by infected DCs (Rothfuchs et al., 2007; van de Veerdonk et al., 2010). Although the beneficiary effects of curdlan have been reported in many diseased conditions such as cancer and leishmaniasis (Leibundgut-Landmann et al., 2008; Ghosh et al., 2013), the anti-*Mtb* activity of curdlan and its mechanism has not been extensively studied.

Induction of nitric oxide synthase (iNOS) is implicated in the control of diverse pathogens including *Mtb* (Chan et al., 1992). Immune cells produce NO through conversion of L-arginine to L-citrulline in the presence of iNOS (Bogdan et al., 2000). Antimicrobial effect of NO has been well established in both murine and human studies (Chan et al., 2001). Few reports have revealed that iNOS activity is controlled by signal transducer and activator of transcription (STAT) signaling pathways; however, the action of STAT-1 in regulating curdlan induced iNOS expression in *Mtb* infected MΦs is not known. In this study, we show that curdlan boosts the functionality of MΦs against *Mtb* and *in vivo* administration of curdlan in *Mtb* infected mice triggered the protective T cell response and reduced *Mtb* burden. Furthermore, curdlan mediated control of *Mtb* survival *via* NO release involved STAT-1 activation. This study demonstrates an important immunotherapeutic role of curdlan against *Mtb* infection.

## Materials and Methods

### Animals

Six-to 7-week old C57BL/6 female mice were obtained from the Animal Facility of CSIR-IMTECH and approved by the Institutional Animal Ethics Committee (IAEC) of CSIR-IMTECH. All the animal experiments and protocols used in the study were approved by the Institutional Animal Ethics Committee (IAEC) of CSIR-IMTECH. The experiments were done in accordance with the National Regulatory Guidelines released by Committee for the Purpose of Control and Supervision of Experiments on Animals (No. 55/1999/CPCSEA), Ministry of Environment and Forest, Government of India.

### Reagents and Antibodies

All standard chemicals and reagents were purchased from Sigma-Aldrich (St. Louis, MO, United States) unless otherwise mentioned. ELISA antibodies, recombinant cytokines, and fluorochrome tagged antibodies for flow cytometry: F4/80-APC, CD11b-PerCP-Cy5.5, CD40-PE-Cy5, CD86-PE, MHC-II-PerCPefluor710, Annexin-FITC, CD4-PB, IL-17-PE, IFN-γ-PE-Cy7, CD62L-FITC, CD44-PerCP-Cy5.5, and CCR7-PECy7 are procured from BD Biosciences (San Diego, CA, United States). Antibodies for western blot analysis against: β-actin, goat anti-rabbit IgG-HRP, and donkey anti-mouse IgG-HRP antibodies were purchased from Santa Cruz Biotechnology (Santa Cruz, CA, United States); iNOS, pSTAT-1 (pY701), STAT-1, pSTAT-3 (pY705), STAT-3, pSTAT-6 (pY641) and STAT-6 antibodies were procured from Cell Signaling Technology (Danvers, MA, United States). Inhibitors used in the experiments such as STAT-1 inhibitor (fludarabine), Syk inhibitor (piceatannol) and iNOS inhibitor (NM, *N*^G^-Monomethyl-L-arginine) are from Calbiochem (Billerica, MA, United States). Curdlan was purchased from InvivoGen (San Diego, CA, United States). All plastic-ware of tissue culture grade was procured from BD Biosciences (Bedford, MA, United States).

### *Mycobacterium tuberculosis* Strains

H37Rv *Mtb* strain was a kind gift from Dr. V. M. Katoch, National JALMA Institute for Leprosy and Other Mycobacterial Diseases, Agra, India. *Mtb* strains were grown in Middlebrook 7H9 broth (Difco) with albumin (10%), dextrose and catalase, glycerol (0.2%) and Tween-80 (0.05%). Aliquots of mid-log phase bacterial cultures were stored at -80°C. Bacterial viability was monitored by plating on 7H11 agar plates *via* colony-forming units (CFUs) assay. Bacterial colonies were enumerated after 21 days (Khan et al., 2016).

### Culture, Infection, and Stimulation of Macrophages (MΦs)

Cells isolated from femurs and tibia of mouse bone marrow were cultured in complete medium consisting of RPMI-1640 (GIBCO Invitrogen Corporation, Grand Island, NY, United States) and 10%-FBS with L929 SN (20%), as a source of M-CSF. The cell cultures were incubated at 5% CO_2_/37°C. The media was replaced on day 3. After 7 days, MΦs were harvested and used for experiments.

For *Mtb* infection experiments, 2 × 10^5^ cells per well (in triplicates) in 48- well tissue culture grade plate were infected with *Mtb* at a MOI of 5 *Mtb*/cell. After 4 h, the cells were extensively washed with PBS (1×) 3–4 times and resuspended in the complete media (antibiotics free RPMI+ 10%-FBS) with amikacin (2 μg/ml) to remove the extracellular bacteria. Amikacin was kept throughout the experiment to kill extracellular bacteria (Khan et al., 2016). Thereafter, cells were stimulated with curdlan (50 μg/ml) for the indicated time points.

For THP-1-derived MΦs culture, THP-1 monocytic cell line was differentiated to MΦs after 16 h of treatment with phorbol 12-myristate 13-acetate (PMA; 25 ng/ml) (Calbiochem, San Diego, CA, United States) (Pahari et al., 2016). Thereafter, cells were washed and then rested for another 16 h prior to curdlan (50 μg/ml) stimulation.

For alveolar MΦs, lungs of mice were washed with 1 ml PBS by flushing several times and the broncho-alveolar lavage was collected. The cells from the solution were pelleted down and were resuspended in DMEM containing 10% FBS for the subsequent infection experiment (Jung et al., 2017).

### Quantification of Cytokines

IL-6, IL-12, IL-10, TNF-α, IL-1β, IL-17, and IFN-γ cytokines were quantified in cell culture SNs by ELISA, as per manufacturer’s instructions (Khan et al., 2016) (BD Pharmingen, San Diego, CA, United States).

### Analysis of MΦs by Flow Cytometry

MΦs were treated with Fc block (anti-CD16/32 antibody) for 30 min followed by incubation with fluorochrome-labeled antibodies (F4/80^+^/CD11b^+^/CD86^+^/CD40^+^/MHC-II^+^) and their isotype-matched control antibodies for another 30 min at 4°C. Later, cells were washed and fixed in PFA (1%). The flow cytometry data were acquired using FACS Aria-II (BD Biosciences, Ashland, OR, United States) (Pahari et al., 2016) and analyzed using DIVA software (BD Biosciences, Ashland, OR, United States). Gating strategy for assessing MΦs phenotype and expression markers through flow cytometry is mentioned in Supplementary Figure S1A. Integrated MFI (iMFI) in the flow cytometry analysis is calculated by multiplying the relative frequency (% positive) of cells expressing a particular marker with the mean fluorescence intensity (MFI) of that population.

### Annexin V and Propidium Iodide (PI) Assay

*Mtb* infected MΦs were stimulated with curdlan (50 μg/ml) for 48 h, and then stained with Annexin V-FITC (5 μl/tube/10^6^ cells) in the dark for 15 min at RT. Thereafter, cells were incubated with 2 μl of PI (50 mg/ml) for 10 min at RT (Pahari et al., 2016). Cells were immediately assessed through flow cytometry using FACS Aria-II (BD Biosciences, Ashland, OR, United States).

### Intracellular Killing Assay

MΦs, 2 × 10^5^ cells per well (in triplicates) in 48- well plate were infected with *Mtb* (MOI of 5) for 4 h followed by extensive washings (3–4 times) with PBS. Later, the cells were suspended in antibiotic-free complete media (RPMI + FBS-10%) with amikacin (2 μg/ml) and treated with curdlan (50 μg/ml) for 48 h. Thereafter, cell culture SNs were removed and cells were made to lyse with saponin (0.1%) and the lysate (100 μl) was plated on mycobacterial media 7H11 agar plates. Bacterial (*Mtb*) colonies were counted after 21 days (Pahari et al., 2016).

In case of experiments with Syk inhibitor, cells were pretreated with Syk inhibitor, piceatannol (20 μg/ml) (Shin et al., 2008) for 45 min prior to *Mtb* infection.

### Antigen and *Mtb* Uptake Assay

For *Mtb* uptake experiments through CFU, MΦs (2 × 10^5^ cells per well in triplicates) in 48- well plate were first stimulated with curdlan (50 μg/ml) for 48 h. It was followed by *Mtb* infection of cells (MOI = 5). After 4 h, cells were washed with PBS and treated for 1 h with amikacin (2 μg/ml) to kill extracellular bacteria. Thereafter, cell SNs were removed and cells were lysed with the saponin (0.1%) and lysate (100 μl) was plated on 7H11 agar plates. Bacterial colonies were enumerated on day 21 (Pahari et al., 2016).

For confocal microscopy experiment, MΦs (3 × 10^5^ cells/well) were stimulated with curdlan for 48 h and then infected with GFP^+^ *Mtb* for 4 h. Thereafter, cells were treated with amikacin for next 1 h followed by washings with cold PBS (1×) and then fixed with PFA (2%) for 15 min. Cells were then kept on poly-L lysine coated coverslips (Khan et al., 2016). The imaging and analysis of intracellular uptake of *Mtb* by MΦs was performed with Nikon A1 confocal laser microscope system (Nikon, Tokyo, Japan). Also, *Z*-stacks were acquired to exclude the possibility of extracellular bacteria.

For dextran antigen uptake test, curdlan stimulated MΦs [4 × 10^5^ cells per well (in triplicates) in 24- well plate] were incubated with dextran antigen labeled with FITC for 30 min at 37°C, or on ice as the control. Dextran uptake was then monitored by flow cytometry.

### Nitric Oxide (NO) Estimation

Supernatants from cell culture were collected and NO was quantified by Griess assay. Equal volume of SNs (50 μl) and Griess reagent (50 μl) was incubated for 5 min/RT (Pahari et al., 2016). Later, absorbance was recorded at 550 nm using NanoDrop spectrophotometer (BioTek, Winooski, VT, United States).

### Quantitative Real-Time PCR (qRT-PCR)

Total RNA was isolated from cells using trizol reagent (Invitrogen, Carlsbad, CA, United States) and RNA purity was determined by NanoDrop spectrophotometer (BioTek, Winooski, VT, United States). Further, 2 μg RNA was used for cDNA synthesis using maxima first cDNA synthesis kit (Thermo Fisher Scientific, Waltham, MA, United States) according to the manufacturer’s protocol. Briefly, RNA was treated with Maxima Reverse Transcriptase enzyme and reaction buffer. Thereafter, RT-PCR and analysis were done on ABI 7500 Fast real-time PCR system (Waltham, MA, United States) at cycling conditions as: 10 min at 95°C, followed by 40 cycles of 15 s at 95°C, 30 s at 60°C, and 60 s at 72°C. The analysis was done using comparative Ct method. β-actin was used as an internal control (Khan et al., 2016). Results are normalized to β-actin and depicted as relative expression (fold change). Primers used in the qRT-PCR are as follows:

**Table 1:** 

*β-actin*	Fwd 5′-AGAGGGAAATCGTGCGTGAC-3′
	Rev 5′-CAATAGTGATGACCTGGCCGT-3′
*Il-6*	Fwd 5′-GAGGATACCACTCCCAACAGACC-3′
	Rev 5′-AAGTGCATCATCATCGTTGTTCATACA-3′
*Il-12*	Fwd 5′-GGAAGCACGGCAGCAGCAGAATA-3′
	Rev 5′-AACTTGAGGGAGAAGTAGGAATGG-3′
*Il-10*	Fwd 5′-GGTTGCCAAGCCTTATCGGA-3′
	Rev 5′-ACCTGCTCCACTGCCTTTGCT-3′
*Inos*	Fwd 5′-AACGGAGAACGTTGGATTTG-3′
	Rev 5′-CAGCACAAGGGGTTTTCTT-3′
*Ifn-*γ	Fwd 5′- CTAAGCAAGGACGGCGAAT -3′
	Rev 5′- TTCCACACTGCACCCACTT-3′
*Tnf-α*	Fwd 5′- GAGCCCCCAGTCTGTGTCCTTCTA-3′
	Rev 5′- CCCCGGCCTTCCAAATAAATACAT-3′
*Dectin-1*	Fwd 5′- AATCCTGTGCTTTGTGGTAG-3′
	Rev 5′- GACTGAGAAAAACCTCCTGTAG-3′
*Arg-1*	Fwd 5′- CCTGAAGGAACTGAAAGGAAA-3′
	Rev 5′- TTGGCAGATATGCAGGGAGT-3′.

### CD4 T Cells Isolation and Co-culture With MΦs

Mice were aerosol challenged with *Mtb* (∼100 CFU). After 21 days of infection, CD4 T cells were isolated from single cell suspension of spleen using BD IMag^TM^ mouse CD4 T lymphocyte enrichment set–DM (BD Biosciences, San Diego, CA, United States) according to the mentioned instruction’s. For co-culture, *Mtb* infected curdlan stimulated MΦs (2 × 10^4^ cells/well) in 96-well plate were co-cultured with CD4 T cells (2 × 10^5^ cells/well) (MΦs: CD4 T cells ratio of 1:10). CD4 T cells were labeled with CFSE (2 μM) prior to co-culture (Rai et al., 2016). PPD (25 μg/ml) was added to the culture to assess *Mtb*-specific T cell response. After 72 h, cells were stained with PE-labeled anti-CD4 antibody. It was followed by analysis of CFSE low CD4 T cell population through flow cytometry. CD4 T cells gating strategy is depicted in Figure 3.

### Western Blotting

MΦs (1 × 10^6^ cells/ml) were harvested and lysed by cytosolic extraction lysis buffer (along with PMSF, protease and phosphatase inhibitor cocktail). Equal concentration of protein in lysates was run on SDS-PAGE, transferred to the PVDF membrane followed by blocking with 5% BSA and probed with iNOS, phospho and non-phospho form of STAT-1, STAT-3, STAT-6, and β-actin (loading control) antibodies (Pahari et al., 2016). Blots were developed by chemiluminescence kit (ECL; Pharmacia-Amersham, Freiburg, Germany) and visualized using ImageQuant LAS 4000 (GE Healthcare, Pittsburgh, PA, United States). Further, images were analyzed by ImageJ analysis software (Fujifilm, New York, NY, United States).

### Therapeutic Strategy

Mice were aerosol challenged with *Mtb* (∼100 CFU). After 21 days of infection, animals were administered curdlan (20 mg/kg of body weight) subcutaneously (s.c.) twice with an interval of 2 weeks. The control group (placebo) was administered with PBS. Anti-TB drug, isoniazid (25 mg/kg body wt. of mice) was orally administrated twice with 0.1% CMC (carboxymethylcellulose) along with the curdlan.

After 7 weeks of infection, animals were sacrificed and *Mtb* burden was assessed in lung and spleen by CFU assay. The weight of the respective organ was taken into consideration while calculating *Mtb* burden (Khan et al., 2016). Bacterial burden was calculated as CFU/0.1 ml × dilution factor × ml/organ = CFU/organ and results are expressed as log_10_.

### Lymphocytes Isolation, T Cells Activation, and Phenotypic Analysis

Mice were perfused with cold PBS containing heparin (100 U/ml) followed by isolation of lungs and spleen. The single cell suspension of tissue was made. Later, cells were treated with ACK lysis buffer to lyse RBCs, washed with PBS (3×) and then resuspended in complete media. Further, cells (2.5 × 10^5^ cells per well in 96-well round bottom plate) were *in vitro* cultured with PPD (25 μg/ml) for 48–72 h to assess the phenotypic markers and intracellular cytokines. For intracellular staining, cells were stimulated with phorbol 12-myristate 13-acetate (PMA) (50 ng/ml) and ionomycin (1 μg/ml) for 4 h followed by the incubation with brefeldin A (5 mg/ml) for another 2 h to block protein transport to the Golgi. Later, cells were stained for the surface marker CD4 and fixed with PFA (4%) for 10 min. The cells were then permeabilized by treatment with saponin buffer (0.2% in 1× PBS) for 15 min/RT. Further, cells were incubated with anti-mouse IFN-γ, and anti-mouse IL-17 antibodies along with their respective isotype-matched controls. Regular washings were carried out after each and every step (Rai et al., 2016). CD4 T cells gating strategy for the intracellular cytokine analysis is depicted in Supplementary Figure S6.

For surface markers analysis, cells were incubated with Fc block for 30 min/4°C and then stained with fluorochrome-labeled antibodies and their matched isotype control antibodies to check the surface expression of CD4, CD44, CD62L, and CCR7 for 30 min at 4°C. Then the stained cells were fixed in PFA (1%) and monitored *via* flow cytometry (Rai et al., 2016). The CD4 T cells gating strategy for the memory phenotype analysis is depicted in Supplementary Figure S7.

Cells were acquired in FACS Aria II and analysis was performed with BD FACS DIVA software.

### Histopathology

Lung specimens were fixed in 10% formalin buffer followed by staining of histological sections with hematoxylin and eosin dye. The microscopic photographs were captured on Olympus IX71 microscope (Olympus, Tokyo, Japan). The images are shown at 40× magnifications.

### NF-κB Activation Analysis

For confocal microscopy, *Mtb* infected MΦs were placed on poly-L-lysine coated coverslips for 15–20 min. The cells were then stimulated with the curdlan (50 μg/ml) for 30 min at 37°C. Later, cells were fixed with PFA (2%) for 10 min, followed by Triton X-100 (0.1%) treatment for 2 min. The samples were then incubated with BSA (2%) for 2 h to block the non-specific sites. Later, cells were incubated with anti-mouse NF-κB p65 Ab (1:400) for 2 h. Subsequently, cells were incubated with Alexa fluor 633-anti-rabbit Ab for 1 h, followed by staining with DAPI dye. Regular washings were performed at each step (Pahari et al., 2016). The cells were imaged through Nikon A1 confocal laser microscope (Nikon, Tokyo, Japan). Data were analyzed using image analysis software, Nikon NIS-AR 4.1 (Nikon, Melville, NY, United States).

For electrophoretic mobility shift assay (EMSA), nuclear extract was prepared from *Mtb* infected and curdlan stimulated MΦs. After 30 min of stimulation, an equal amount of nuclear extract (3 μg) from each sample was incubated for 20 min/37°C in water bath with [P^32^] end labeled duplex oligonucleotides that contains binding site for NF-κB. The DNA–protein complexes were resolved on a native PAGE-gel (7%) by electrophoresis (Pahari et al., 2016). The gel was dried and exposed to screen at RT for 6–12 h and scanned by phosphor-imager scanning screen (Fujifilm, FLA-5000, Tokyo, Japan).

### Statistical Analysis

One-way non-parametric ANOVA test and Student’s *t*-test were used for analysis of the multiple and two groups, respectively. Data were analyzed with Graph Pad Prism 6 software. *P*-values of <0.05 were considered significant.

## Results

### Curdlan Restricts Mycobacterial Growth in MΦs

**FIGURE 1:**
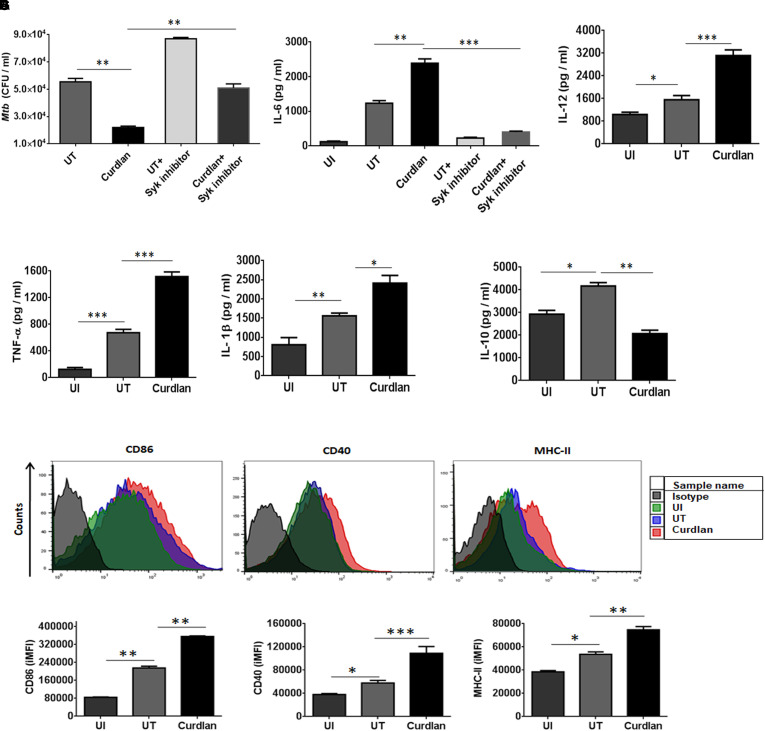
Curdlan induces the activation and maturation of MΦs. Bone marrow-derived MΦs were infected with *Mtb* (at MOI of 5) and treated with curdlan (50 μg/ml) for 48 h. **(A,B)** Cells were pretreated with Syk inhibitor, piceatannol (20 μg/ml) for 45 min prior to infection. After 48 h, **(A)** cells were lysed and plated on the 7H11 agar plates. The *Mtb* CFUs were enumerated after 21 days. Further, cell culture SNs were assessed for the production of cytokines such as **(B)** IL-6; **(C)** IL-12; **(D)** TNF-α; **(E)** IL-1β; and **(F)** IL-10 by ELISA. Additionally, **(G)** cells were assessed for the expression of CD86, CD40, and MHC-II with fluorescent tagged antibodies and their isotype controls. Representative flow cytometry histogram shows overlay plots and bar graphs signify integrated mean fluorescence intensity (iMFI) values. Data expressed as mean ± SD are representative from three independent experiments. ^∗^*p* ≤ 0.05, ^∗∗^*p* ≤ 0.01, ^∗∗∗^*p* ≤ 0.001. UI (uninfected), MΦs not infected with *Mtb*; UT (untreated), *Mtb* infected MΦs; Curdlan, *Mtb* infected MΦs stimulated with curdlan.

Initially, we examined the effect of curdlan on *Mtb* survival in bone marrow-derived MΦs. Dose titration experiment showed optimum secretion of IL-6 by MΦs at a dose of 50 μg/ml of curdlan and was found to be non-cytotoxic (Supplementary Figures S1B, S2). *Mtb* infected MΦs treated with curdlan in comparison to untreated control, exhibited significant (*p* ≤ 0.01) decrease in intracellular *Mtb* growth as seen in low CFU counts concomitant with the increase in IL-6 cytokine (*p* ≤ 0.01) release in cell SNs (Figure 1A,B). Further, to define the specificity of the involved pathway, we inhibited the Syk kinase, as curdlan signals in a dectin-1/Syk dependent manner (Brown, 2006). Inhibitor of Syk, piceatannol substantially reduced the stimulatory effect of curdlan in infected MΦs as seen in the impaired clearance of *Mtb* (*p* ≤ 0.01) and IL-6 (*p* ≤ 0.001) production (Figure 1A,B). The ability of curdlan to restrict *Mtb* growth was also confirmed using THP-1 derived MΦs and alveolar macrophages through CFU assay (Supplementary Figures S3A,B). It is interesting to note that curdlan exerts its *Mtb* killing effect optimally at 48 h of treatment (Supplementary Figure S3C). Curdlan induces elevated levels of proinflammatory cytokines IL-12 (*p* ≤ 0.001), TNF-α (*p* ≤ 0.001) and IL-1β (*p* ≤ 0.05) (Figure 1C–E), while anti-inflammatory IL-10 secretion was found to be reduced (*p* ≤ 0.01) in culture SNs of *Mtb* infected MΦs versus untreated control (Figure 1F). Additionally, we found upregulated expression of activation markers such as CD86, CD40, and MHC-II on MΦs after curdlan treatment through flow cytometry (Figure 1G). The gating strategy for assessing macrophage population is depicted in Supplementary Figure S1A. Moreover, curdlan elicits higher expression of genes such as dectin-1 (receptor for curdlan) along with IL-6 and TNF-α that are involved in protective immune response while suppressive Arg-1 was found to be downregulated in *Mtb* infected MΦs (Supplementary Figure S4).

### Curdlan Enhances MΦs Phagocytosis Ability

**FIGURE 2:**
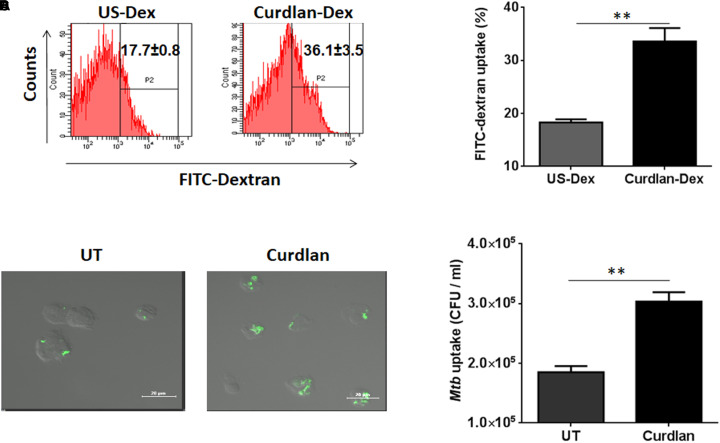
Stimulation through curdlan enhances the phagocytic ability of MΦs. **(A–D)** MΦs were stimulated with the curdlan (50 μg/ml) for 48 h. Thereafter, **(A,B)** cells were incubated with dextran-FITC particles for 30 min at 37°C and then uptake was examined by flow cytometry. Insets of flow cytometry plots and bar graph show percentage of positive cells gated on macrophage population, US-Dex: unstimulated macrophages incubated with dextran-FITC, Curdlan-Dex, curdlan stimulated macrophages incubated with dextran-FITC. **(C)** Curdlan stimulated cells were infected with *Mtb*^GFP^ [green] and after 4 h, phagocytosis was monitored by confocal microscopy. **(D)** Curdlan activated cells were infected with *Mtb* H37Rv and 4 h post infection, uptake was evaluated by CFU assay. UT: *Mtb* infected MΦs; Curdlan: *Mtb* infected and curdlan stimulated MΦs. Data represented as mean ± SD and representative from two independent experiments. ^∗∗^*p* ≤ 0.01.

Next, we sought to determine the uptake ability of curdlan stimulated MΦs. Thus, we stimulated the MΦs with curdlan followed by their incubation with Dextran-FITC. Flow cytometry data showed enhanced (*p* ≤ 0.01) dextran antigen uptake, relative to unstimulated MΦs (Figure 2A,B). The gating strategy is same as shown in Supplementary Figure S1A. For *Mtb* phagocytic assay, MΦs were first stimulated with curdlan followed by *Mtb* infection. Thereafter, GFP-*Mtb* uptake was assessed by confocal microscopy (Figure 2C). Further, these results were confirmed by significant increase (*p* ≤ 0.01) of *Mtb* uptake as observed through CFU assay (Figure 2D). This data suggests that curdlan has the capacity to improve phagocytosis of *Mtb* by MΦs.

### Curdlan Stimulated MΦs Induce Better *Mtb* Antigen-Specific CD4 T Cell Proliferation and Th1/Th17 Immune Response

**FIGURE 3:**
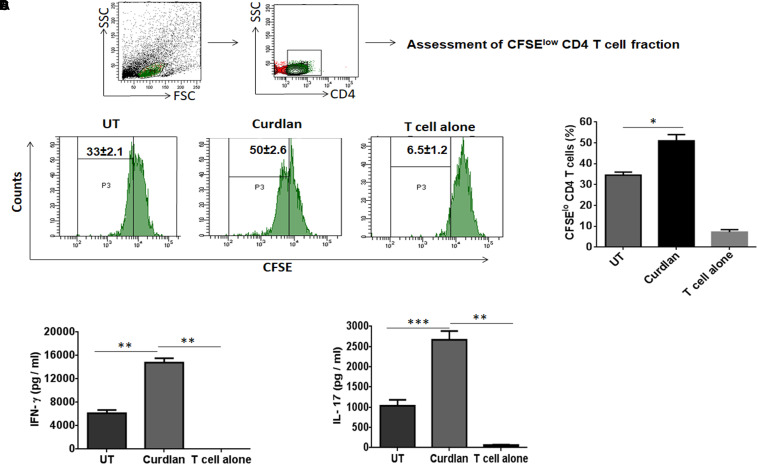
Stimulation of *Mtb* infected MΦs with curdlan activate *Mtb* antigen-specific T cells. MΦs were infected with *Mtb* (MOI = 5) and stimulated with curdlan (50 μg/ml) for 48 h, followed by their co-culture with CFSE (2 μM) labeled CD4 T cells (MACS sorted from spleen of *Mtb* infected mice) in the presence of PPD (25 μg/ml) at a ratio of 1:10. **(A,B)** After 72 h, CD4 T cells proliferation was monitored by flow cytometry. First gate (P1) was made in live lymphocyte zone followed by gating on CD4 T cells to assess cell proliferation. Data in the inset of flow cytometry plot and bar graph indicate percentage of proliferating CD4 T cells (CFSE^lo^). Further, **(C,D)** IFN-γ and IL-17 were measured in the culture SNs by ELISA. UT, *Mtb* infected MΦs (not stimulated with curdlan); Curdlan: *Mtb* infected and curdlan stimulated MΦs. Data represented as mean ± SD are representative from two independent experiments. ^∗^*p* ≤ 0.05, ^∗∗^*p* ≤ 0.01, ^∗∗∗^*p* ≤ 0.001.

One of the important attributes of antigen presenting cell is to activate T cells (Khan et al., 2012). Thus, we investigated the stimulatory potential of *Mtb* infected curdlan stimulated MΦs to activate CD4 T cells (isolated from spleen of *Mtb* infected mice) in the presence of PPD (*Mtb* antigen). Curdlan treatment of infected MΦs resulted in the better proliferation of CD4 T cells in comparison to untreated macrophages as determined by flow cytometry analysis (Figure 3A,B). In addition, these CD4 T cells secreted higher levels of IFN-γ (*p* ≤ 0.01) and IL-17 (*p* ≤ 0.001) cytokines as detected in culture SNs by ELISA. This represents the induction of Th1 and Th17 response, respectively, by curdlan activated MΦs (Figure 3C,D).

### *In vivo* Therapeutic Efficacy of Curdlan Against TB

**FIGURE 4:**
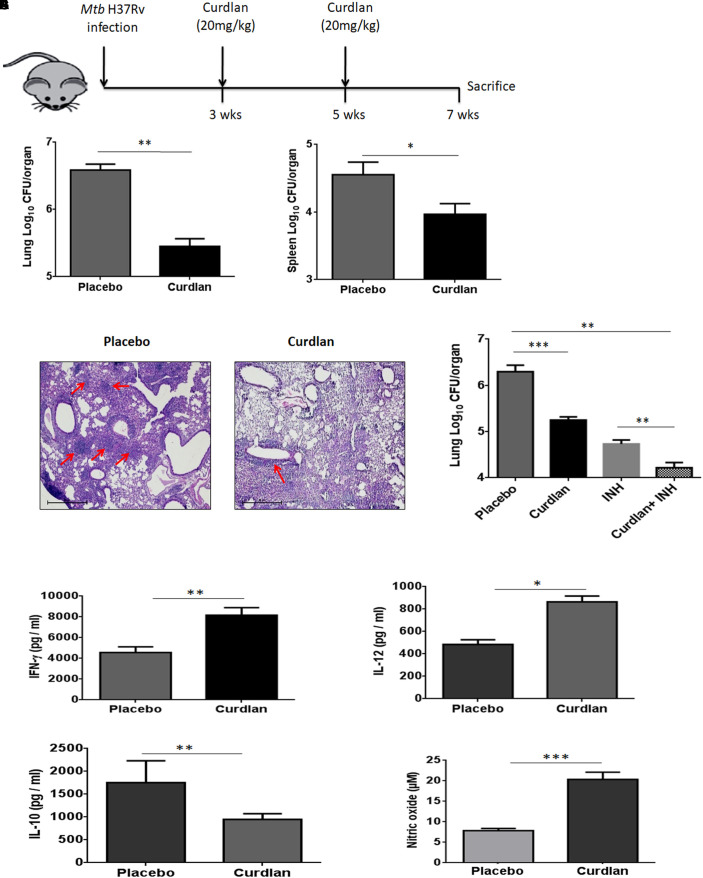
Administration of curdlan reduces the *Mtb* burden in the mouse model of TB. **(A)** Mice were aerosol challenged with *Mtb* (∼100 CFU) followed by subcutaneous administration of curdlan (20 mg/kg) after 21 days of infection. Curdlan was administered twice after the gap of 2 weeks. After 7 weeks of *Mtb* infection, animals were sacrificed and **(B,C)** *Mtb* burden in **(B)** lung and **(C)** spleen was enumerated by CFU assay. **(D)** Photomicrographs (40×) of hematoxylin and eosin stained lung sections; arrows depict the number of granulomas per field. **(E)** *Mtb* infected mice were administered curdlan in combination with anti-TB drug [isoniazid (INH), 25 mg/kg body wt] twice as described above. After 7 weeks post infection, mycobacterial load in the lungs were enumerated by CFU assay. **(F–I)** Lung cells from placebo and curdlan treated mice were cultured in the presence of PPD (25 μg/ml) for 48 h and thereafter supernatant was assessed for the levels of **(F)** IFN-γ; **(G)** IL-12; and **(H)** IL-10 by ELISA. Further, **(I)** Nitric oxide levels were quantified by Griess assay. Data are represented as mean ± SD of two independent experiments. For every experiment, consisting of 4–5 mice/group, cells from each mouse were plated in triplicate wells, and their average values were used to calculate the mean ± SD. ^∗^*p* ≤ 0.05, ^∗∗^*p* ≤ 0.01, ^∗∗∗^*p* ≤ 0.001. Placebo, *Mtb* infected mice administered with PBS; Curdlan, *Mtb* infected mice treated with curdlan; INH, Isoniazid.

Next, we assessed the therapeutic effect of curdlan in the experimental model of TB. After 21 days of *Mtb* infection, curdlan was administered subcutaneously (s.c.) in mice twice after the gap of 2 weeks (Figure 4A). Strikingly, curdlan therapy significantly reduced the *Mtb* burden in the lungs (*p* ≤ 0.01) and spleen (*p* ≤ 0.05) as evident by the decline in CFU counts (Figure 4B,C). Further, histological analysis of lung tissue also showed improved pathology and less granulomatous lesions as compared to placebo control (Figure 4D). Interestingly, we also observed that immunization of *Mtb* infected mice with curdlan in combination with isoniazid (INH) considerably (*p* < 0.01) reduced the *Mtb* burden in lungs. Thus, curdlan administration along with INH imparted better protection against *Mtb* (Figure 4E).

In addition, curdlan treatment led to higher levels of IFN- γ (*p* ≤ 0.01), IL-12 (*p* ≤ 0.05), and NO (*p* ≤ 0.001) whereas IL-10 (*p* ≤ 0.01) secretion was found to be reduced in culture SNs of lung cells (Figure 4F–I). We further validated the cytokines and NO production at mRNA level through qRT-PCR (Supplementary Figure S5).

### Curdlan Therapy in Mice Induces *Mtb* Antigen-Specific IFN-γ and IL-17 Producing CD4 T Cells

**FIGURE 5:**
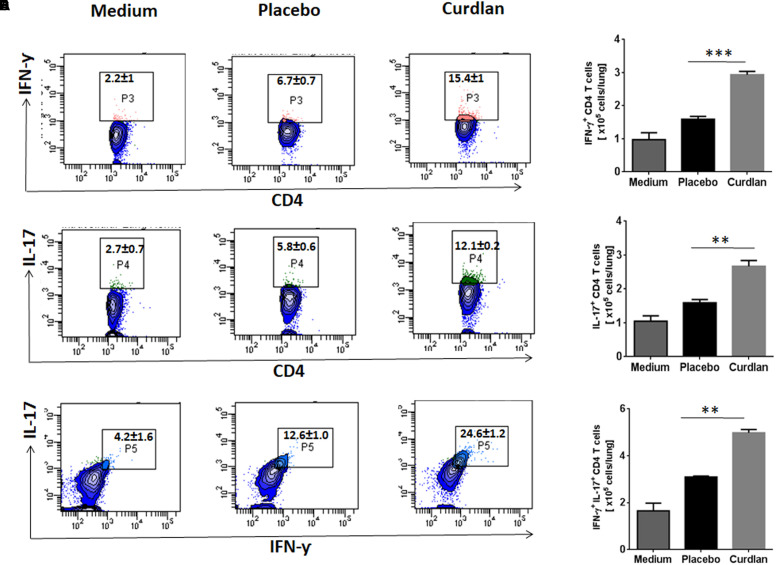
Immunotherapy with curdlan elicits Th1 and Th17 immune response in *Mtb* challenged mice. *Mtb* infected mice were immunized with curdlan as described in legend to Figure 4. After 7 weeks post infection, lung cells were isolated and cultured with PPD (25 μg/ml) for 72 h. Later, intracellular staining was performed for examining the **(A,B)** IFN-γ^+^, **(C,D)** IL-17^+^, and **(E,F)** IFN-γ^+^/IL-17^+^ cells by flow cytometry. Contour plots insets represent the percentage of IFN-γ^+^ (pink colored), IL-17^+^ (green) and IFN-γ^+^/IL-17^+^ (light blue) cells gated on CD4 T cell population (indicated in blue) and bar graphs shows the absolute number of positive cells per lung. Data are shown as mean ± SD and are representative from two independent experiments. For each experiment, consisting of 4–5 mice/group, cells from individual mouse were plated in triplicate wells, and their average values were used to calculate the mean ± SD. ^∗∗^*p* ≤ 0.01, ^∗∗∗^*p* ≤ 0.001. Medium, unstimulated cells; Placebo, *Mtb* infected mice administered with PBS; Curdlan, *Mtb* infected mice treated with curdlan.

CD4 T cells secreting IFN-γ and IL-17 serve as main effector cells during *Mtb* infection (Flynn et al., 1993; Gopal et al., 2014). We next evaluated the impact of curdlan administration on *Mtb* antigen-specific T cell response against *Mtb*. As shown in Figure 5A–D, lungs cells of mice treated with curdlan exhibited both higher percentage and absolute numbers of IFN-γ^+^ (*p* ≤ 0.001) and IL-17^+^ (*p* ≤ 0.01) CD4 T cells after *in vitro* stimulation with PPD as compared to placebo control indicating effective generation of Th1 and Th17 immunity. Further, the population of polyfunctional CD4 T cells co-expressing both IFN-γ and IL-17 (*p* ≤ 0.01) was also increased in these mice as determined by flow cytometry (Figure 5E,F). The gating strategy for flow cytometry analysis of IFN-γ and IL-17 producing CD4 T cells is depicted in Supplementary Figure S6.

### Curdlan Treatment *in vivo* Enhances CD4 T Cell Proliferation and Memory Response

**FIGURE 6:**
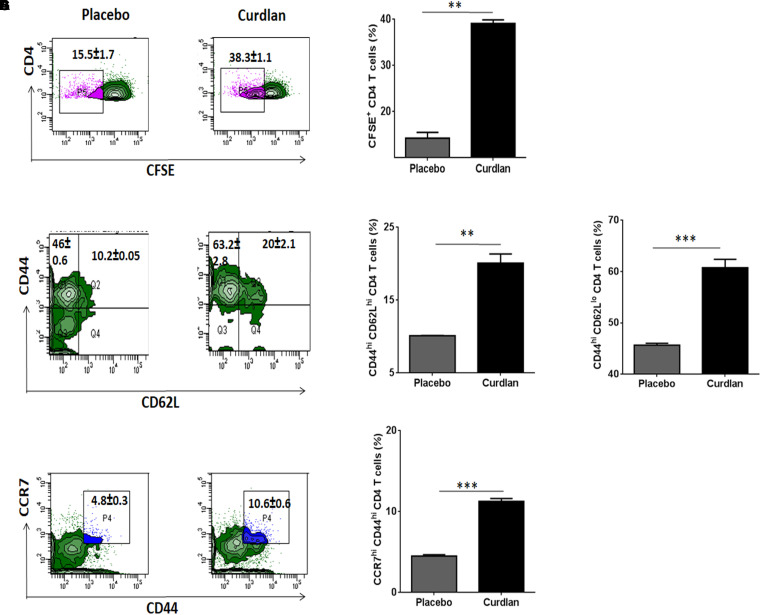
Curdlan administration evokes T cell proliferation and generates enduring memory response in *Mtb* infected mice. *Mtb* challenged animals were immunized with curdlan as stated in legend to Figure 4. After 7 weeks post challenge, lung cells were harvested and **(A,B)** CFSE (2 μM) labeled prior to *in vitro* culture with PPD (25 μg/ml) for 72 h to monitor CD4 T cell proliferation by flow cytometry. Additionally, **(C–G)** lung cells suspension were stained with fluorochrome tagged antibodies and their isotype controls as described in section “Materials and Methods” and examined for **(C,D)** Central (CD4^+^/CD44^hi^/CD62L^hi^); **(C,E)** Effector memory phenotype (CD4^+^/CD44^hi^/CD62L^low^) and **(F,G)** CD4^+^/CD44^hi^/CCR7^hi^ cells gated on CD4 T cells by flow cytometry analysis. Numbers in the inset of flow cytometry plot and bar graphs indicates the percentage of positive cells. The data are represented as mean ± SD and representative from two independent experiments. Each experimental group was composed of 4–5 mice. ^∗∗^*p* ≤ 0.01, ^∗∗∗^*p* ≤ 0.001. Placebo, *Mtb* infected mice administered with PBS; Curdlan, *Mtb* infected mice treated with curdlan.

*Mtb* is known to impair the proliferation capability of antigen-specific CD4 T cells, crucial for host immunity (Canaday et al., 2001). After curdlan administration in *Mtb* infected mice, lung lymphocytes were isolated (7 weeks post *Mtb* infection), CFSE (2 μM) labeled and *in vitro* stimulated with PPD (25 μg/ml) for 72 h to assess the CD4 T cell proliferation through flow cytometry. Interestingly, these mice exhibited enhanced (*p* ≤ 0.01) ability of CD4 T cells to proliferate in comparison to placebo control (Figure 6A,B).

Another hallmark of effective and long-term protection is the generation of memory T cells. They provide an efficient immune response on pathogen re-exposure (Gourley et al., 2004). *In vivo* administration of curdlan resulted in a considerably increased frequency of central (CD44^hi^/CD62L^hi^) (*p* ≤ 0.01) and effector memory (CD44^hi^/CD62L^low^) (*p* ≤ 0.001) CD4 T cell pool (Figure 6C–E). Further, chemokine receptor CCR7 (C-C chemokine receptor type 7) is involved in the migration of memory T cells to peripheral organs (Ohl et al., 2004). Thus, we next evaluated the migratory potential of activated CD4 T cells in the lungs of animals. Intriguingly, we observed elevated (*p* ≤ 0.001) population of CD44^hi^/CCR7^hi^ CD4 T cells in curdlan treated mice versus placebo control (Figure 6F,G). The gating strategy for memory phenotype analysis of CD4 T cells is depicted in Supplementary Figure S7.

Moreover, we also found substantial increase of central memory cells represented as CD44^hi^CD62L^hi^CCR7^hi^ and effector memory (CD44^hi^CD62L^lo^CCR7^lo^) CD4 T cells in lungs of mice with curdlan therapy (Supplementary Figure S7B).

### Curdlan Elicits Generation of Nitric Oxide in *Mtb* Infected MΦs

**FIGURE 7:**
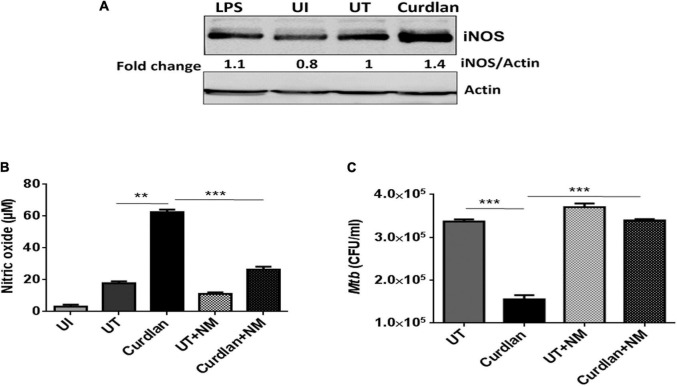
Curdlan activated MΦs augments nitric oxide production. MΦs were infected with *Mtb* (MOI = 5) for 4 h, **(A)** cells were then stimulated with curdlan (50 μg/ml) and after 18 h, iNOS protein level was assessed in cell lysates through western blotting; **(B,C)** Infected cells were pretreated for 1 h with iNOS inhibitor (*N*-monomethyl-L-arginine; 20 μM) prior to stimulation with curdlan for 48 h. Thereafter, **(B)** secretion of NO was monitored in cell culture SNs by Griess method; further, **(C)** cells were lysed and plated on 7H11 agar plates to determine *Mtb* survival by CFU assay. UI, MΦs not infected with *Mtb*; UT, *Mtb* infected MΦs; Curdlan, *Mtb* infected and curdlan stimulated MΦs; NM, *N*-monomethyl-L-arginine. The data shown as the mean ± SD are representative from two independent experiments. ^∗∗^*p* < 0.01, ^∗∗∗^*p* < 0.001.

Next, we were interested to decipher the mechanism involved in curdlan mediated killing of *Mtb*. The expression of iNOS and NO production has been shown to play a pivotal role in *Mtb* killing (Chan et al., 1992). Thus, we treated *Mtb* infected MΦs with curdlan and assessed the expression of iNOS by western blot. Interestingly, curdlan induced higher levels of iNOS relative to untreated MΦs (Figure 7A). This observation is in line with the increased (*p* ≤ 0.01) NO production in curdlan stimulated MΦs (Figure 7B). Further, the involvement of NO was confirmed by inhibiting its synthesis with iNOS inhibitor, *N*-mono-methyl L-arginine (NM). The curdlan mediated increase in NO secretion and *Mtb* killing was impaired upon NM treatment (Figure 7B,C). These results indicate that NO plays a crucial role in curdlan induced clearance of *Mtb* in MΦs.

### Curdlan Activates STAT-1 and NF-κB in *Mtb* Infected MΦs

**FIGURE 8:**
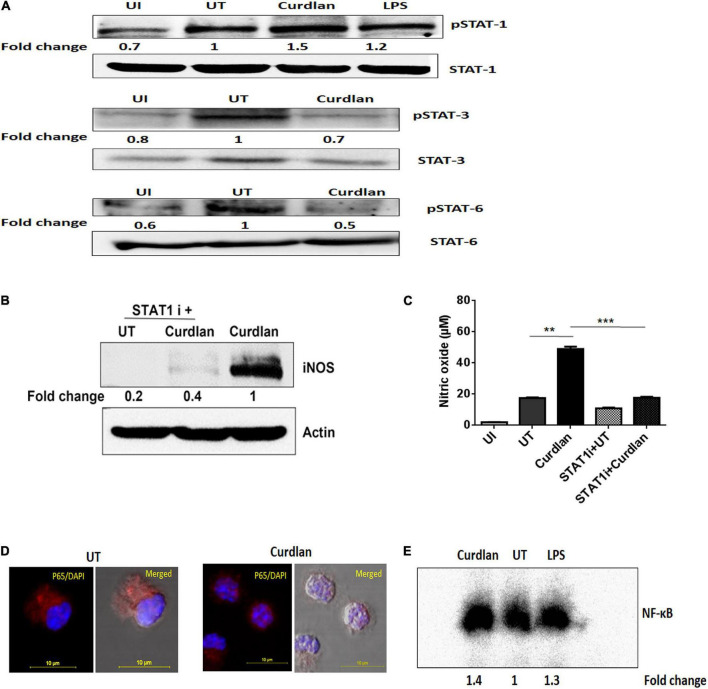
Curdlan activates STAT-1 and NF-κB in *Mtb* infected MΦs. MΦs were infected with *Mtb* (MOI of 5) for 4 h followed by treatment with curdlan (50 μg/ml). **(A)** After 15–30 min of curdlan stimulation, cell lysates were prepared and analyzed for pSTAT-1, STAT-1, pSTAT-3, STAT-3, pSTAT-6, STAT-6 by western blot. β-actin was used as loading control. **(B,C)** Infected MΦs were pretreated with STAT-1 inhibitor (STAT1 i) fludarabine (50 μM) for 1 h prior to curdlan stimulation for 18 h (to assess iNOS) and 48 h (to examine nitric oxide release), **(B)** iNOS expression in cell lysates by western blot; blots are representative of two independent experiments. Further, **(C)** nitric oxide level in cell culture SNs was assessed by Griess assay; data shown as mean ± SD are representative from two independent experiments, each performed in triplicates, ^∗∗^*p* < 0.01, ^∗∗∗^*p* < 0.001. Further, **(D,E)** Infected MΦs were stimulated with curdlan for 30 min. Thereafter, **(D)** nuclear translocation of NF-κB in MΦs (p65 subunit) was examined through confocal microscopy; p65 subunit [red]; nucleus stained with DAPI [blue]. **(E)** Nuclear extract of MΦs depicts NF-κB activation by EMSA assay as fold change compared to untreated. Data is representative of two independent experiments. UI, MΦs not infected with *Mtb*; UT, *Mtb* infected MΦs; Curdlan, *Mtb* infected and curdlan stimulated MΦs; STAT1 i + UT, *Mtb* infected MΦs treated with STAT1 inhibitor; STAT1 i + Curdlan, *Mtb* infected MΦs treated with STAT1 inhibitor prior to curdlan stimulation; LPS, lipopolysaccharide (2 μg/ml).

We further assessed the activation of signaling pathways in curdlan stimulated MΦs during *Mtb* infection. Given that STAT-1 has been reported to be involved in the suppression of pathogens including *Mtb* (Najjar and Fagard, 2010). Additionally, Tyr701 phosphorylation of STAT-1 is related to the expression of iNOS (Heitmeier et al., 1999), thus we were interested to examine STAT pathway involvement in curdlan triggered iNOS expression in *Mtb* infected MΦs. We performed western blot analysis using cell lysate of *Mtb* infected curdlan treated and untreated MΦs. Strikingly, we found that the level of pSTAT-1 was increased in curdlan stimulated MΦs versus untreated control (Figure 8A). Likewise, we observed a reduction of phosphorylated STAT-6 and STAT-3 in these MΦs. We ascertained the role of STAT-1 in curdlan mediated iNOS expression and NO generation with fludarabine (a specific inhibitor of STAT1; STAT1 i). As shown in Figure 8B,C, MΦs treated with STAT-1 inhibitor prior to curdlan stimulation displayed impaired iNOS expression and NO secretion.

Moreover, we observed considerable activation and translocation of NF-κB into the nucleus of *Mtb* infected and curdlan treated MΦs as seen by confocal microscopy and also corroborated this result by EMSA (Figure 8D,E). These data indicate the significant role of STAT-1 and NF-κB pathways in curdlan-mediated anti-*Mtb* effect.

## Discussion

Treatment of TB is extremely challenging due to drug-associated side effects and the rise of resistant *Mtb* strains (Kaufmann, 2013). This imperatively demands to develop innovative therapies for better management and treatment of TB. Recently, host-directed therapies involving immunomodulators have gained attention in restricting *Mtb* infection (Khan et al., 2016; Pahari et al., 2016, 2017). Innate immunity receptors *viz.* TLRs, CLRs, NLRs, and RLRs plays a central role in host defense against the array of pathogens (Kawai and Akira, 2011). Immunomodulators that trigger signaling through innate receptors present on MΦs can be utilized against *Mtb* (Pahari et al., 2018). Curdlan is a biological response modifier that stimulates dectin-1 receptor on MΦs and other myeloid cells (Goodridge et al., 2009). It has been approved by FDA, with proven beneficial effects in leishmania, cancer, bacterial and viral infections (Ghosh et al., 2013). There is lack of studies depicting mechanistic role of curdlan in imparting immunity against *Mtb*. Thus, we investigated *in vitro* and *in vivo* efficacy of curdlan during *Mtb* infection. Further, the underlying mechanism and therapeutic potential of curdlan to be used as host-directed therapy in TB was demonstrated.

In order to generate protective immune response against *Mtb*, optimum expression of costimulatory molecules such as CD86, CD80, and CD40 on MΦs is very crucial for the activation of T cells (Aderem and Underhill, 1999; Harris and Ronchese, 1999; Reis e Sousa, 2004; Joffre et al., 2009) along with the production of pro-inflammatory cytokines (Domingo-Gonzalez et al., 2016). Curdlan triggered activation of MΦs against *Mtb* can be attributed to: (i) upregulation of MHC-II, CD40, and CD86 expression; (ii) augmented secretion of IL-12, IL-1β, and TNF-α, while the decline in suppressive IL-10; (iii) enhanced capacity to phagocytose and kill *Mtb*; (iv) improved ability to activate and proliferate CD4 T cells. Further, *in vivo* curdlan administration resulted in: (i) increased Th1 and Th17 immune response with the expanded pool of memory CD4 T cells in lungs; (ii) declined *Mtb* survival in lungs and spleen along with improved lung pathology in *Mtb* infected mice. Previously, studies have highlighted the beta glucans mediated activation of antigen presenting cells that cause expansion and differentiation of T cells against tumors (Leibundgut-Landmann et al., 2008). It is worth to mention here that our study also demonstrates the generation of *Mtb* specific CD4 T cell response by curdlan activated macrophages. The limitations of the study include lack of dectin-1 knockdown or knockout models. Dectin-1 agonist curdlan have been reported to work through the Syk pathway (Brown, 2006). Thus, we have examined the specificity of curdlan acting *via* Syk dependent dectin-1 pathway by piceatannol (Syk inhibitor) (Shin et al., 2008) that resulted in impairment of curdlan mediated *Mtb* killing in macrophages. However, the possibility of Syk-independent effects of piceatannol cannot be ruled out and impact of more specific Syk inhibitors such as R406 needs future investigation.

CD4 T cells play an indispensable role in conferring host defense against *Mtb* (Canaday et al., 2001). Th1 cells and Th17 cells play a crucial role in mounting a protective immune response against *Mtb* (Flynn et al., 1993; Gopal et al., 2014). Interestingly, in line with the previous studies (Hsieh et al., 1993; LeibundGut-Landmann et al., 2007), we observed that curdlan induced the secretion of Th1 and Th17-polarizing cytokines, such as IL-12 and IL-1β, respectively, in *Mtb* infected macrophages. Accordingly, we found expanded pool of Th1 cells and Th17 cells in the animals with curdlan therapy. This is consistent with the substantial decline in *Mtb* load in lungs, as well as prevented dissemination of *Mtb* to the spleen. Recently, polyfunctional T cells have been reported to impart better protection against the infection (Caccamo et al., 2010; Rai et al., 2016). Furthermore, the presence of polyfunctional IFN-γ^+^/IL-17^+^ CD4 T cells was evident in *Mtb* infected animals with curdlan treatment. The establishment of effective memory T cell response is necessary to impart long-term protection (Lindenstrom et al., 2009; Singh et al., 2010). Our results suggested better memory T cell response against *Mtb* as seen by the enhanced pool of central and effector memory CD4 T cells. Thereby, indicating a crucial impact of curdlan in improving the immunological memory.

The mechanism involved in curdlan mediated clearance of *Mtb* is unknown. The role of NO in host defense against *Mtb* has been reported earlier (Chan et al., 1992). Here, we demonstrate that stimulation of MΦs with curdlan induced higher expression of iNOS (involved in NO production). Inhibition of NO generation impaired the curdlan mediated *Mtb* killing.

Further, STATs are crucial in regulating the functionality and polarization of MΦs (Ohmori and Hamilton, 2001; Kernbauer et al., 2012). Interestingly, previous reports suggest that STAT-1 deficiency is linked to impaired anti-*Mtb* immunity while its activation results in protective MΦ phenotype that possesses anti-microbial activity. On the other hand, STAT-3 and STAT-6 phosphorylation are prominent in suppressive MΦs that are unable to restrict *Mtb* survival (Sica and Mantovani, 2012; Lim et al., 2016). Moreover, STAT-1 and STAT-3 reciprocally regulate each other (Costa-Pereira et al., 2002; Qing and Stark, 2004). Our study is consistent with these findings, we show that curdlan activated MΦs exhibit increased STAT-1 phosphorylation while pSTAT-3 and pSTAT-6 levels were reduced indicating sufficient ability of MΦs to restrict *Mtb* growth. This is in accordance with the elevated level of IL-12, TNF-α, iNOS (protective factors) and reduced expression of IL-10 and Arg-1 (immunosuppressive factors) upon curdlan treatment. Thus, STAT-1 phosphorylation sustains the activation of curdlan stimulated MΦs. Strikingly, studies have described the regulation of iNOS expression by phosphorylation of STAT-1 (Lowenstein et al., 1993; Heitmeier et al., 1999). We hypothesize that STAT-1 binds to the promoter sequences of *Inos* gene and enhances the expression of iNOS and therefore the generation of NO. In line with this, we validate our findings by blocking STAT-1 with the specific STAT-1 inhibitor fludarabine. Interestingly, it abrogated the iNOS expression and NO secretion induced by curdlan. Thus, curdlan induced STAT-1 activation is required for the iNOS expression and NO production. Further, another important transcription factor, NF-κB plays a crucial role in controlling *Mtb* infection by inducing expression of many proinflammatory cytokines and protective immune mediators (Davis et al., 2005; Fallahi-Sichani et al., 2012). We show that curdlan stimulation in *Mtb* infected MΦs resulted in NF-κB activation which is also consistent with the observed induction of IL-12, TNF-α, IL-6, and iNOS.

**FIGURE 9:**
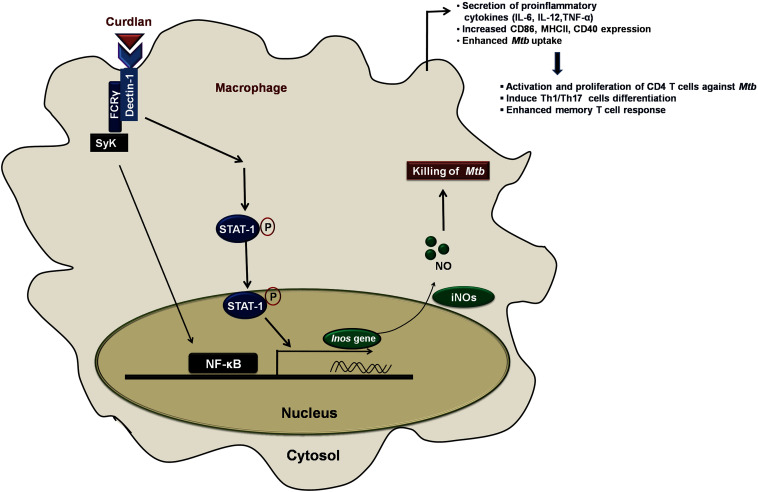
Curdlan induced signaling pathways and events in *Mtb* infected MΦs. This proposed model illustrates the signaling events involved in restricting the *Mtb* growth. Curdlan binds to its receptor, dectin-1 and induces the STAT-1 phosphorylation in *Mtb* infected MΦs. This leads to the pSTAT-1 mediated activation of iNOS gene to generate NO, which has antimicrobial properties to restrict the *Mtb* survival. Subsequently, there is activation and translocation of NF-κB into the nucleus. These signaling pathways activate and boost the function of MΦs (increased proinflammatory cytokines, bacterial uptake, expression of costimulatory molecules) which further elicit the protective effector and memory T cell response against *Mtb*.

In summary, we have demonstrated that anti-*Mtb* activity of curdlan is through activation of STAT-1 and NF-κB pathway activation that resulted in NO production and thus restricting the *Mtb* survival (Figure 9). This approach of boosting the host immune system with immunomodulators such as curdlan may prove to be an important therapeutic approach to treat TB.

## Ethics Statement

Mice were obtained from the Animal Facility of CSIR-IMTECH and approved by the Institutional Animal Ethics Committee (IAEC) of CSIR-IMTECH. All the animal experiments and protocols used in the study were approved by the Institutional Animal Ethics Committee (IAEC) of CSIR-IMTECH. The experiments were done in accordance with the National Regulatory Guidelines released by Committee for the Purpose of Control and Supervision of Experiments on Animals (No. 55/1999/CPCSEA), Ministry of Environment and Forest, Government of India.

## Author Contributions

JA and SN conceived the idea and designed work. SN, SP, DD, and NK performed the experiments. JA and SN analyzed and interpreted the data and wrote the manuscript.

## Conflict of Interest Statement

The authors declare that the research was conducted in the absence of any commercial or financial relationships that could be construed as a potential conflict of interest.

## Abbreviations

CCR7: C-C chemokine receptor type 7
CFU: colony forming unit
DCs: dendritic cells
FDA: food and drug administration
HIV: human immunodeficiency virus
iNOS: inducible NO synthase
LPS: lipopolysaccharide
MΦs: macrophages
MOI: multiplicity of infection
*Mtb*: *Mycobacterium tuberculosis*
NF-κB: nuclear factor kappa B
NM: *N*-mono-methyl L-arginine
NO: nitric oxide
PFA: paraformaldehyde
PPD: purified protein derivative
PRRs: pattern recognition receptors
SNs: supernatants
STAT-1 i: signal transducer and activator of transcription-1 inhibitor
Syk: spleen tyrosine kinase
TB: tuberculosis
UI: uninfected
UT: untreated
