# Corrigendum: Curdlan Limits *Mycobacterium tuberculosis* Survival Through STAT-1 Regulated Nitric Oxide Production

**DOI:** 10.3389/fmicb.2022.924981

**Published:** 2022-05-18

**Authors:** Shikha Negi, Susanta Pahari, Deepjyoti Kumar Das, Nargis Khan, Javed N. Agrewala

**Affiliations:** ^1^Immunology Division, CSIR - Institute of Microbial Technology, Chandigarh, India; ^2^Immunology Division, Texas Biomedical Research Institute, San Antonio, TX, United States; ^3^Department of Microbiology and Immunology, McGill University, Montreal, QC, Canada; ^4^Department of Biomedical Engineering, Indian Institute of Technology Ropar, Rupnagar, India

**Keywords:** macrophages, curdlan, iNOS, T cells, host-directed therapy, tuberculosis

In the original article, there was a mistake in the legend for Figure 8 as published. We have corrected Figure 8B and the legend has also been corrected. The correct Figure 8 and legend appears below.

In the original article, there was a mistake in Figure 7A as published. Figure 7A, the actin panel was a cut and paste duplication error of STAT1 in Figure 8A. We have now inserted the appropriate actin control and calculated the fold change accordingly. The corrected Figure 7 appears below.

**Figure 7:**
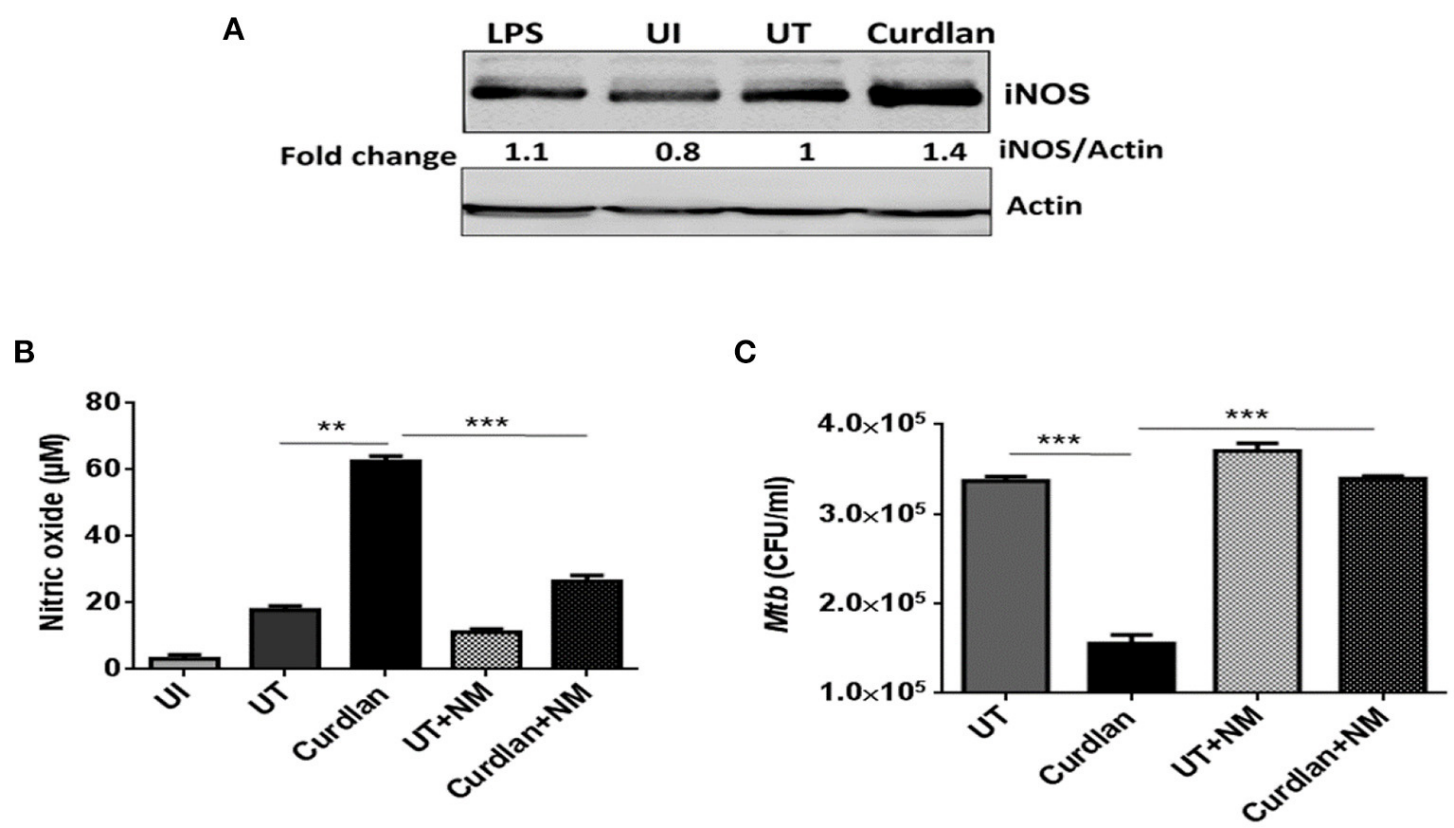
Curdlan activated MΦs augments nitric oxide production. MΦs were infected with *Mtb* (MOI = 5) for 4 h, **(A)** cells were then stimulated with curdlan (50 μg/ml) and after 18 h, iNOS protein level was assessed in cell lysates through western blotting; **(B,C)** Infected cells were pretreated for 1 h with iNOS inhibitor (*N*-monomethyl-L-arginine; 20 μM) prior to stimulation with curdlan for 48 h. Thereafter, **(B)** secretion of NO was monitored in cell culture SNs by Griess method; further, **(C)** cells were lysed and plated on 7H11 agar plates to determine *Mtb* survival by CFU assay. UI, MΦs not infected with *Mtb*; UT, *Mtb* infected MΦs; Curdlan, *Mtb* infected and curdlan stimulated MΦs; NM, *N*-monomethyl-L-arginine. The data shown as the mean ± SD are representative from two independent experiments. ^**^p < 0.01, ^***^p < 0.001.

**Figure 8:**
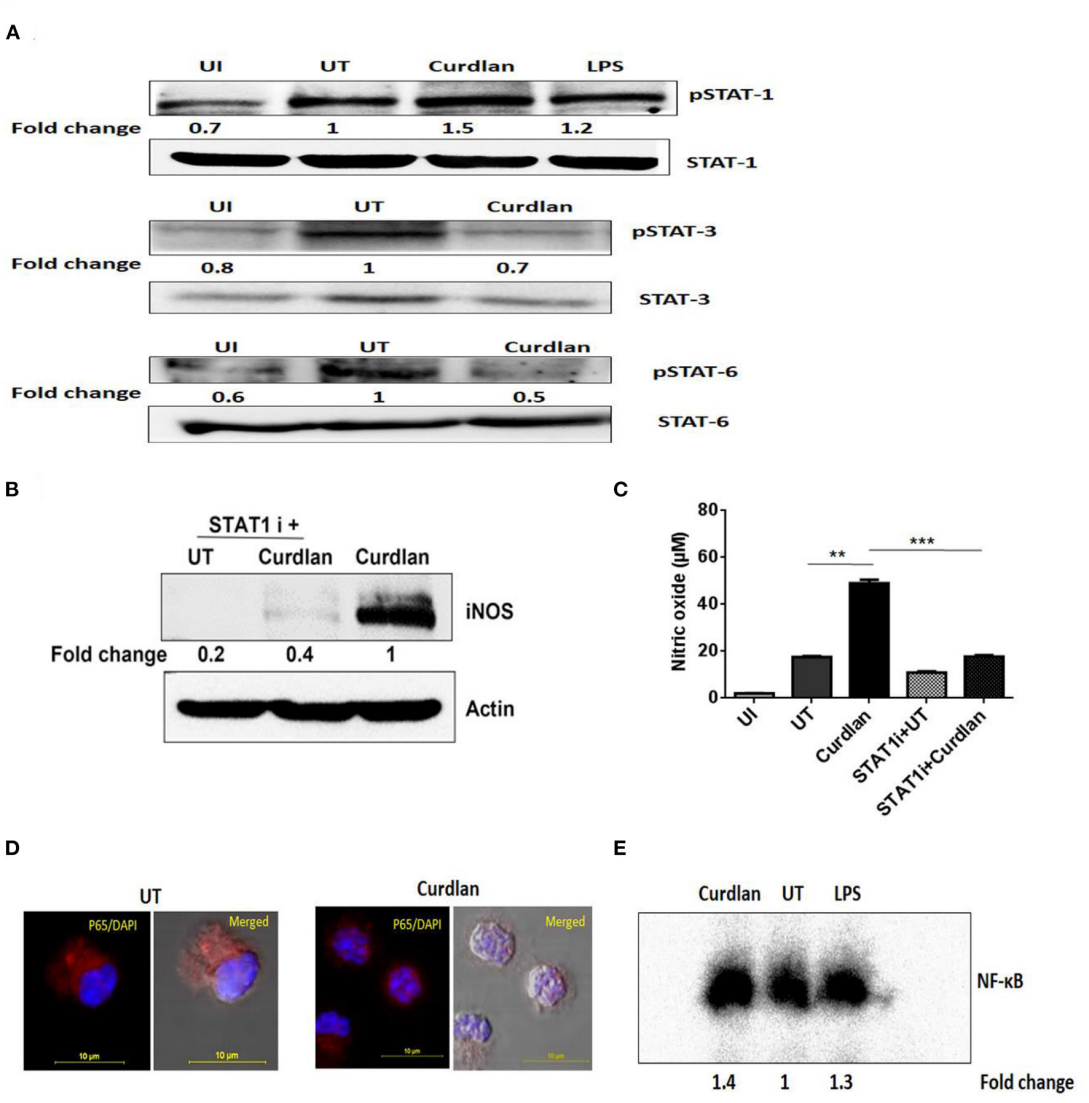
Curdlan activates STAT-1 and NF-κB in *Mtb* infected MΦs. MΦs were infected with *Mtb* (MOI of 5) for 4 h followed by treatment with curdlan (50 μg/ml). **(A)** After 15–30 min of curdlan stimulation, cell lysates were prepared and analyzed for pSTAT-1, STAT-1, pSTAT-3, STAT-3, pSTAT-6, STAT-6 by western blot. β-actin was used as loading control. **(B,C)** Infected MΦs were pretreated with STAT-1 inhibitor (STAT1 i) fludarabine (50 μM) for 1 h prior to curdlan stimulation for 18 h (to assess iNOS) and 48 h (to examine nitric oxide release), **(B)** iNOS expression in cell lysates by western blot; blots are representative of two independent experiments. **(C)** Nitric oxide level in cell culture SNs was assessed by Griess assay; data shown as mean ± SD are representative from two independent experiments, each performed in triplicates, **p < 0.01, ***p < 0.001. Further, **(D,E)** Infected MΦs were stimulated with curdlan for 30 min. Thereafter, **(D)** nuclear translocation of NF-κB in MΦs (p65 subunit) was examined through confocal microscopy; p65 subunit [red]; nucleus stained with DAPI [blue]. **(E)** Nuclear extract of MΦs depicts NF-κB activation by EMSA assay as fold change compared to untreated. Data is representative of two independent experiments. UI, MΦs not infected with *Mtb*; UT, *Mtb* infected MΦs; Curdlan, *Mtb* infected and curdlan stimulated MΦs; STAT1 i + UT, *Mtb* infected MΦs treated with STAT1 inhibitor; STAT1 i + Curdlan, *Mtb* infected MΦs treated with STAT1 inhibitor prior to curdlan stimulation; LPS, lipopolysaccharide (2 μg/ml).

The authors apologize for this error and state that this does not change the scientific conclusions of the article in any way. The original article has been updated.

## Publisher's Note

All claims expressed in this article are solely those of the authors and do not necessarily represent those of their affiliated organizations, or those of the publisher, the editors and the reviewers. Any product that may be evaluated in this article, or claim that may be made by its manufacturer, is not guaranteed or endorsed by the publisher.

