# Supplementary material for: Curdlan Limits Mycobacterium tuberculosis Survival Through STAT-1 Regulated Nitric Oxide Production
Source: Front Microbiol. 2019 May 28;10:1173. doi: 10.3389/fmicb.2019.01173 (PMC6547911; doi:10.3389/fmicb.2019.01173)

**Supplementary Figures**

**Supplementary Figure 1**. (A) Gating strategy for flow cytometry analysis of bone marrow-derived macrophages (MΦs). First, gate (P1) was made on cells excluding debris on a FSC *vs* SSC dot plot; thereafter F4/80 and CD11b positive population (P2) was gated to further analyse the expression of activation and phenotypic markers on MΦ population. The purity of F4/80^+^ CD11b^+^ MΦs was >90% as determined *via* flow cytometry. (B) IL-6 secretion in culture supernatants of MΦs stimulated with various doses of curdlan was examined by ELISA, data shown as mean±SD from two independent experiments.


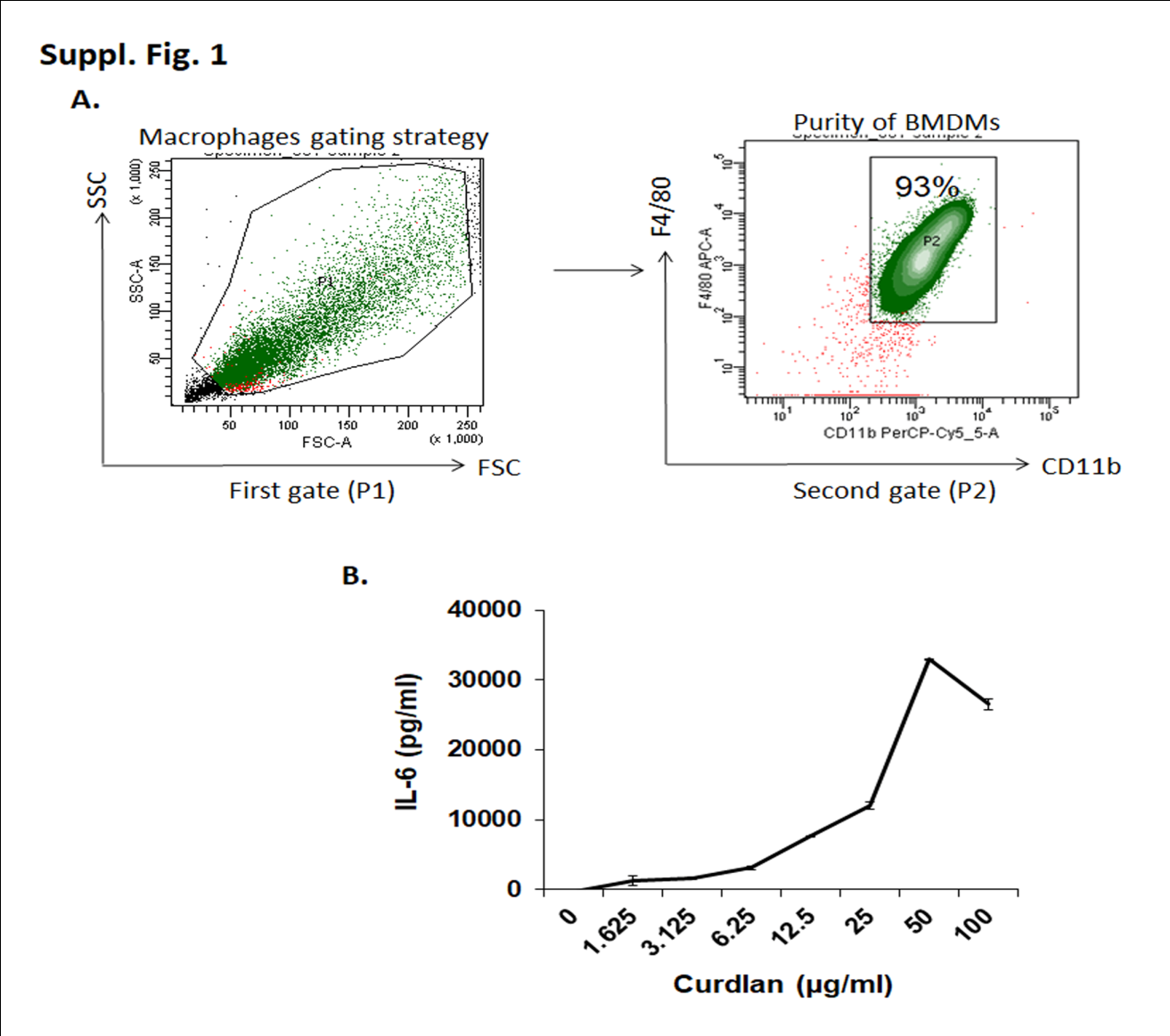


**Supplementary Figure 2**. MΦs were infected with H37Rv for 4 h at MOI of 1:5 and then stimulated with curdlan (50 µg/ml) for 48 h. Cells were stained with annexin V-FITC, followed by propidium iodide (PI) and assessed by flow cytometry. Number in the insets of dot plots indicates the percentage of Annexin V and PI positive cells. Data expressed as mean±SD and representative from two independent experiments. UI (uninfected): MΦs not infected with *Mtb*; UT (untreated): *Mtb* infected MΦs; Curdlan: *Mtb* infected MΦs stimulated with curdlan.


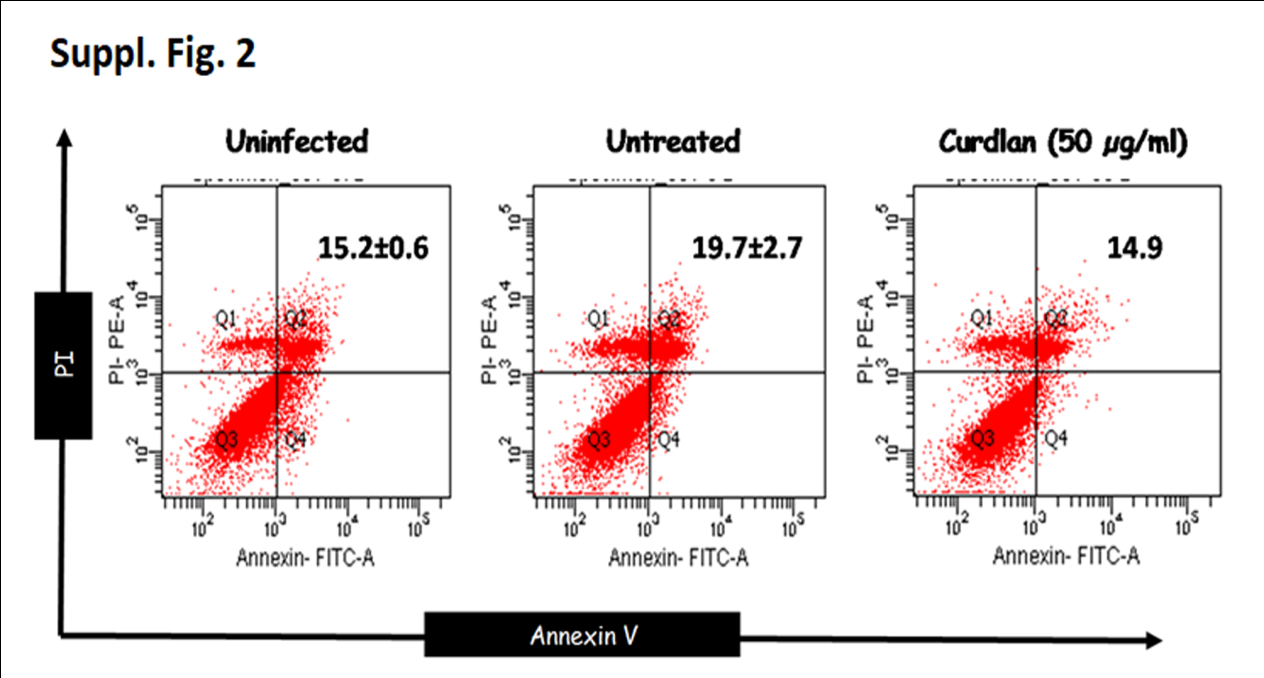


**Supplementary Figure 3**. (A) THP-1 derived MΦs and (B) Alveolar macrophages were infected with *Mtb* (MOI of 5) for 4 h and stimulated with curdlan (50 µg/ml) for 48 h. Thereafter, cells were lysed and plated on 7H11 agar plates. (C) Bone marrow derived macrophages were infected with *Mtb* and stimulated with curdlan followed by CFU analysis (as described in methods) at the indicated time points. CFU counts were seen after 21 d. Data represent mean±SD from two independent experiments. **p≤0.01. UT (untreated): *Mtb* infected MΦs; Curdlan: *Mtb* infected MΦs stimulated with curdlan.


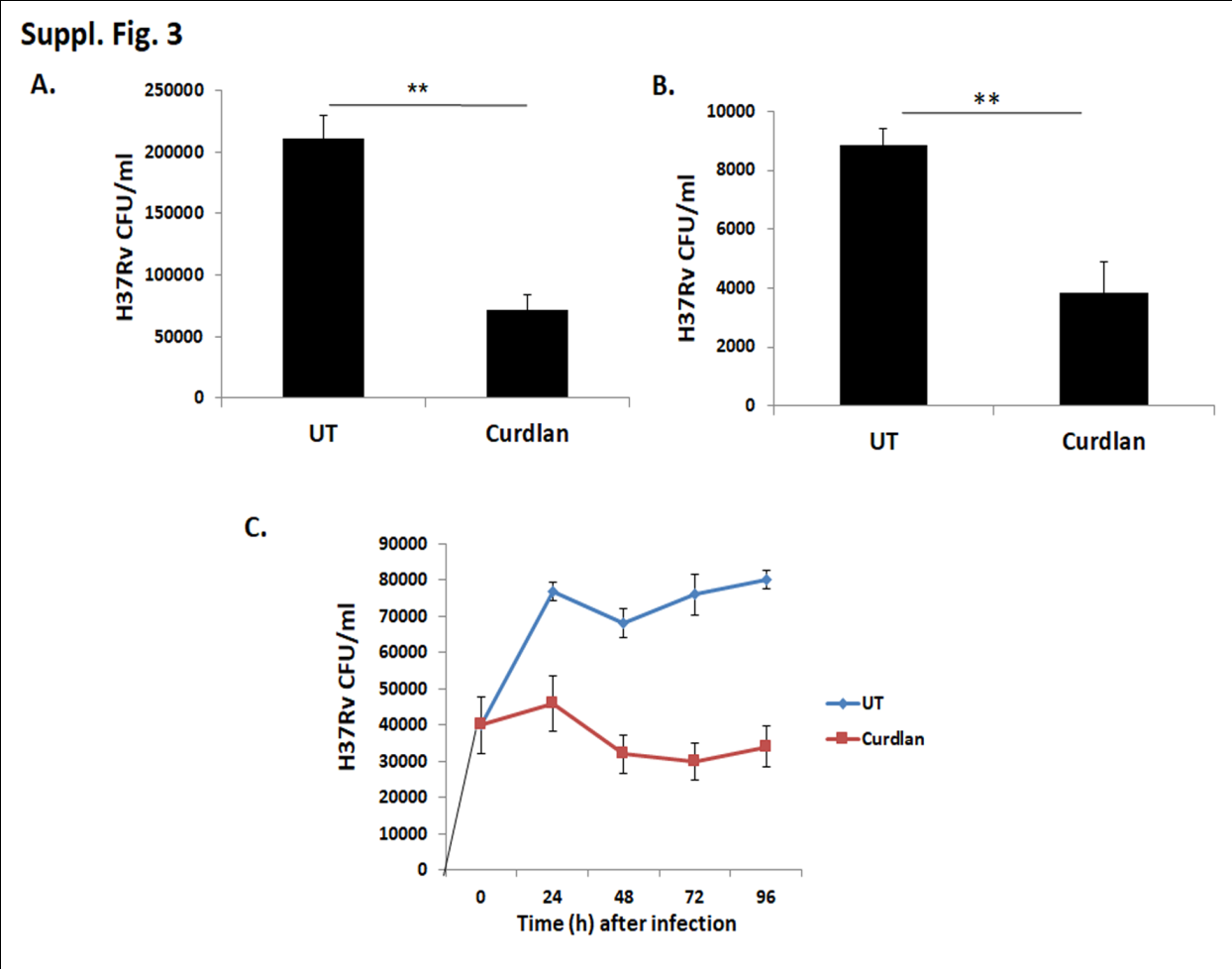


**Supplementary Figure 4**. MΦs infected with *Mtb* (MOI of 5), stimulated with curdlan (50 µg/ml) for 48 h and then lysed with TRIzol for RNA isolation and cDNA synthesis as described in Methods. Thereafter, expression of (A) Dectin-1; (B) IL-6; (C) TNF-α; and (D) Arg-1 gene was assessed by qRT-PCR as fold change normalised to β-actin. Data represented as mean±SD from two independent experiments. *p≤0.05, **p≤0.01, ***p≤0.001. UT (untreated): *Mtb* infected MΦs; Curdlan: *Mtb* infected MΦs stimulated with curdlan.


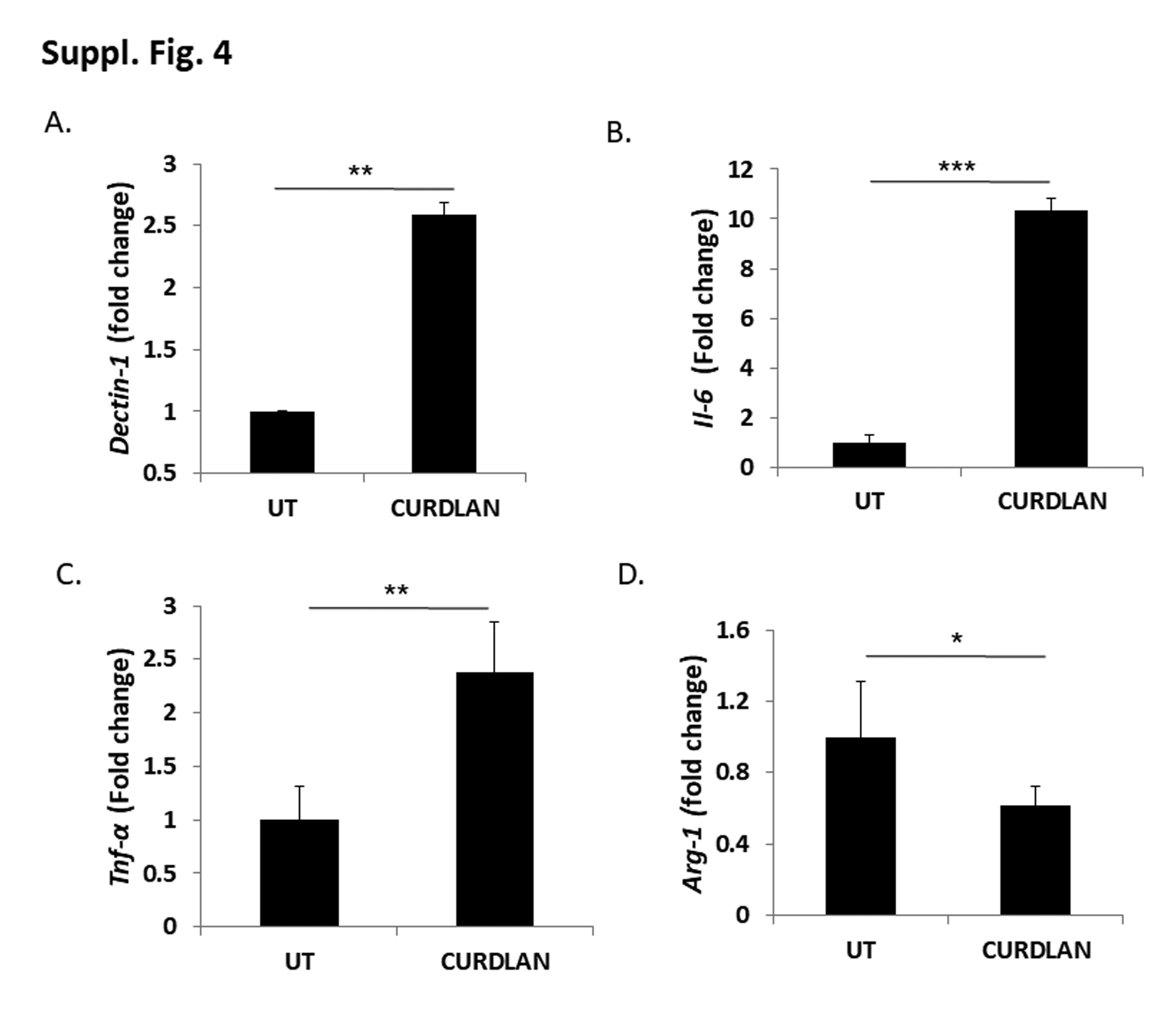


**Supplementary Figure 5.** Administration of curdlan enhances protective cytokines and nitric oxide mRNA levels in the lung of *Mtb* infected mice. Mice were aerosol challenged with *Mtb* (~100 CFU) followed by subcutaneous administration of curdlan (20 mg/kg) after 21 d of infection. Curdlan was administered twice after the gap of 2 weeks. After 7 wk of *Mtb* infection, animals were sacrificed and lung cells were cultured in the presence of PPD (25 µg/ml) for 48 h and thereafter were assessed for the mRNA levels of (A) IL-12; (B) IFN-γ; (C) IL-10; (D) iNOS genes by qRT-PCR and depicted as fold change normalized to β-actin; and Data are represented as mean±SD from two independent experiments. *p≤0.05, ***p≤0.001; n=4-5 animals/group. Placebo: *Mtb* infected mice administered with PBS; Curdlan: *Mtb* infected mice treated with curdlan.


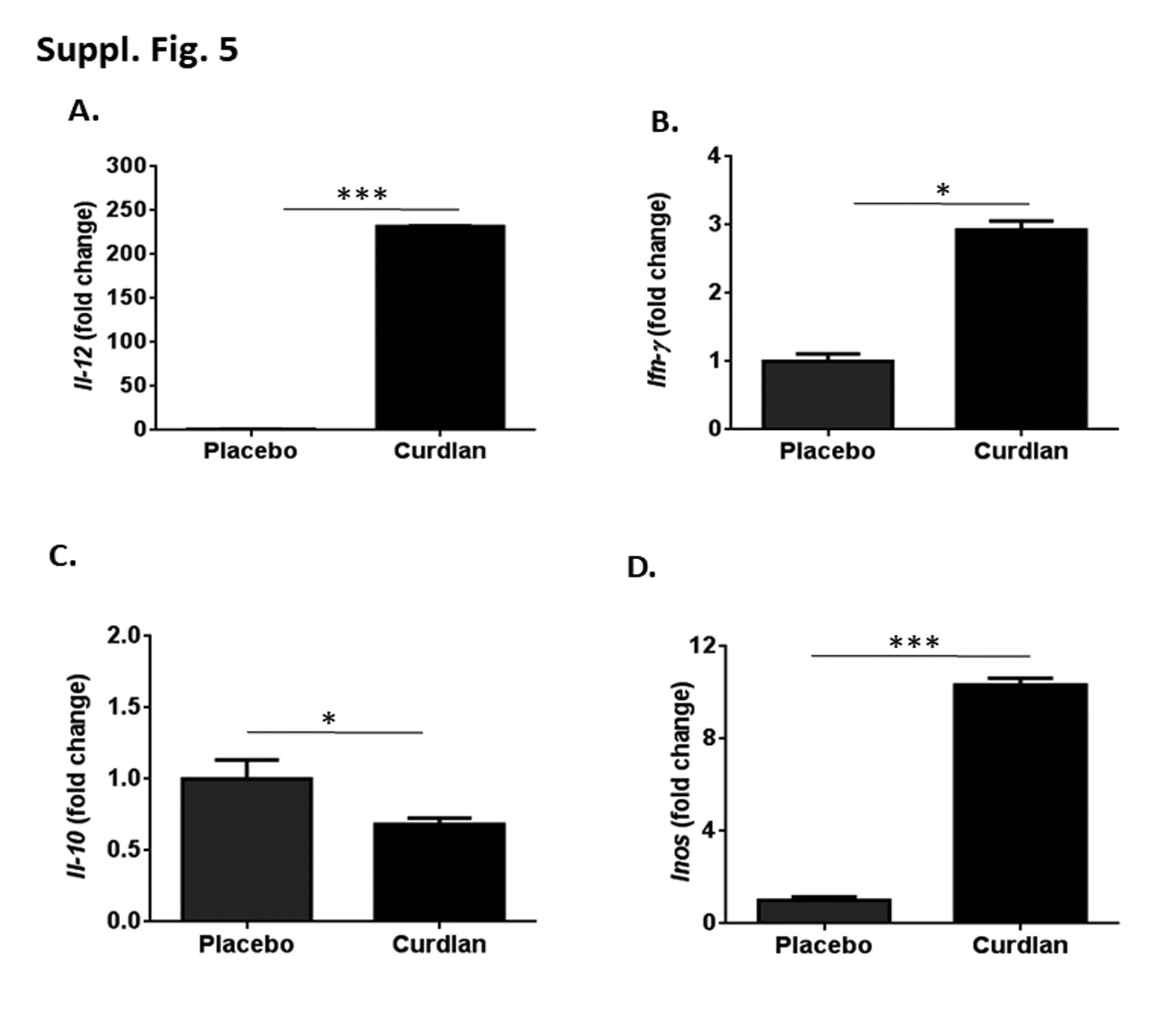


**Supplementary Figure 6.** Gating strategy for monitoring the expression of IFN-γ and IL-17 on CD4 T cells. Lung lymphocytes were stained with fluorochrome labeled antibodies to IFN-γ and IL-17 along with their isotype controls and assessed by flow cytometry. First, P1 gate was made on live lymphocyte zone on the basis of forward-scatter (FSC) and side-scatter (SSC) analysis followed by the second gate on SSC-A and CD4 T cells (P2 gate). Further, analysis of IFN-γ^+^, IL-17^+^ and IFN-γ^+^ IL-17^+^ population was done on these P2 gated cells only. Data is representative from two independent experiments with n=5 mice/group.


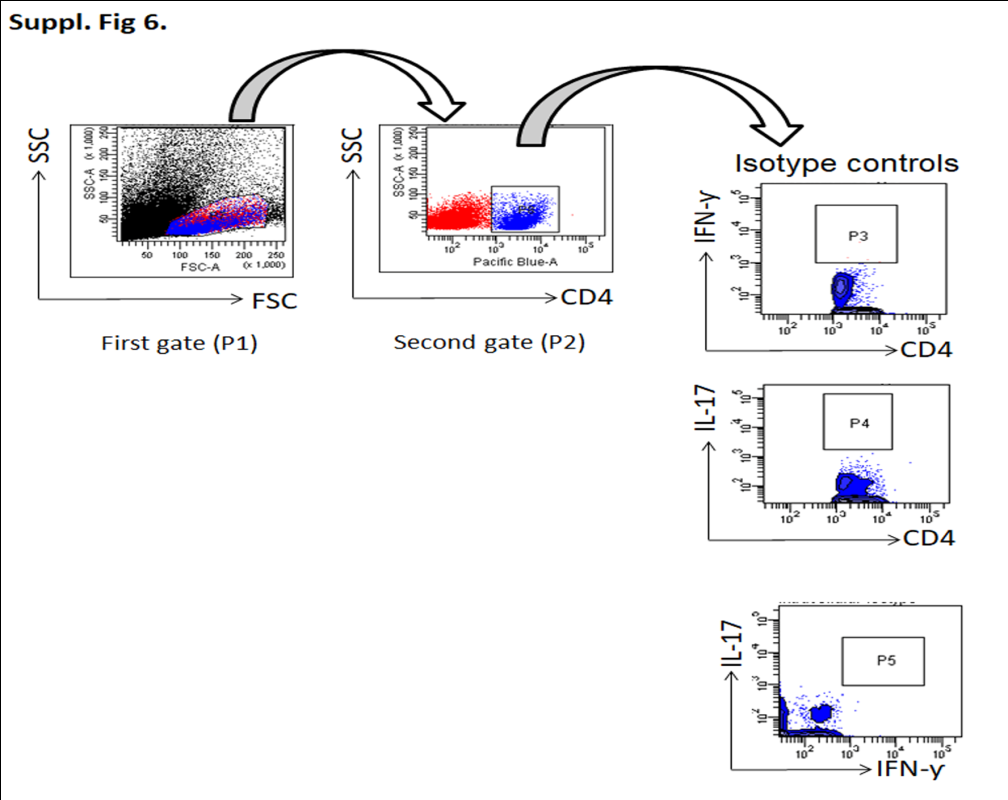


**Supplementary Figure 7.** Gating strategy for assessing the expression of memory markers on CD4 T cells. (A) The primary P1 gate was made on the live lymphocytes zone on the basis of forward-scatter (FSC) and side-scatter (SSC) excluding the debris. Next, the secondary gate (P2) was specified on the SSC-A/CD4 T cells zone. The expression of CD44, CD62L and CCR7 was monitored on the P2 zone. For the analysis of CD62L^lo^ CCR7^lo^ CD44^hi^ (T_EM_) and CD62L^hi^ CCR7^hi^ CD44^hi^ (T_CM_) population, CD44^hi^ population was gated on either CD62L^lo^ CCR7^lo^ or CD62L^hi^ CCR7^hi^ sub-populations. Isotype controls did not show any positive population. (B) *Mtb* infected animals were immunized with curdlan as described in legend to Fig. 4. After 7 wk post challenge, lung lymphocytes were harvested and stained with fluorochrome tagged antibodies and isotype controls for monitoring the central (CD62L^hi^ CCR7^hi^ CD44^hi^) and effector memory (CD62L^lo^ CCR7^lo^ CD44^hi^) phenotype. Numbers in the inset of flow cytometry plot indicates the percentage of positive cells, data expressed as mean±SD and representative from two independent experiments with n=5 mice/group. Placebo: *Mtb* infected mice treated with PBS; Curdlan: *Mtb* infected mice treated with curdlan.


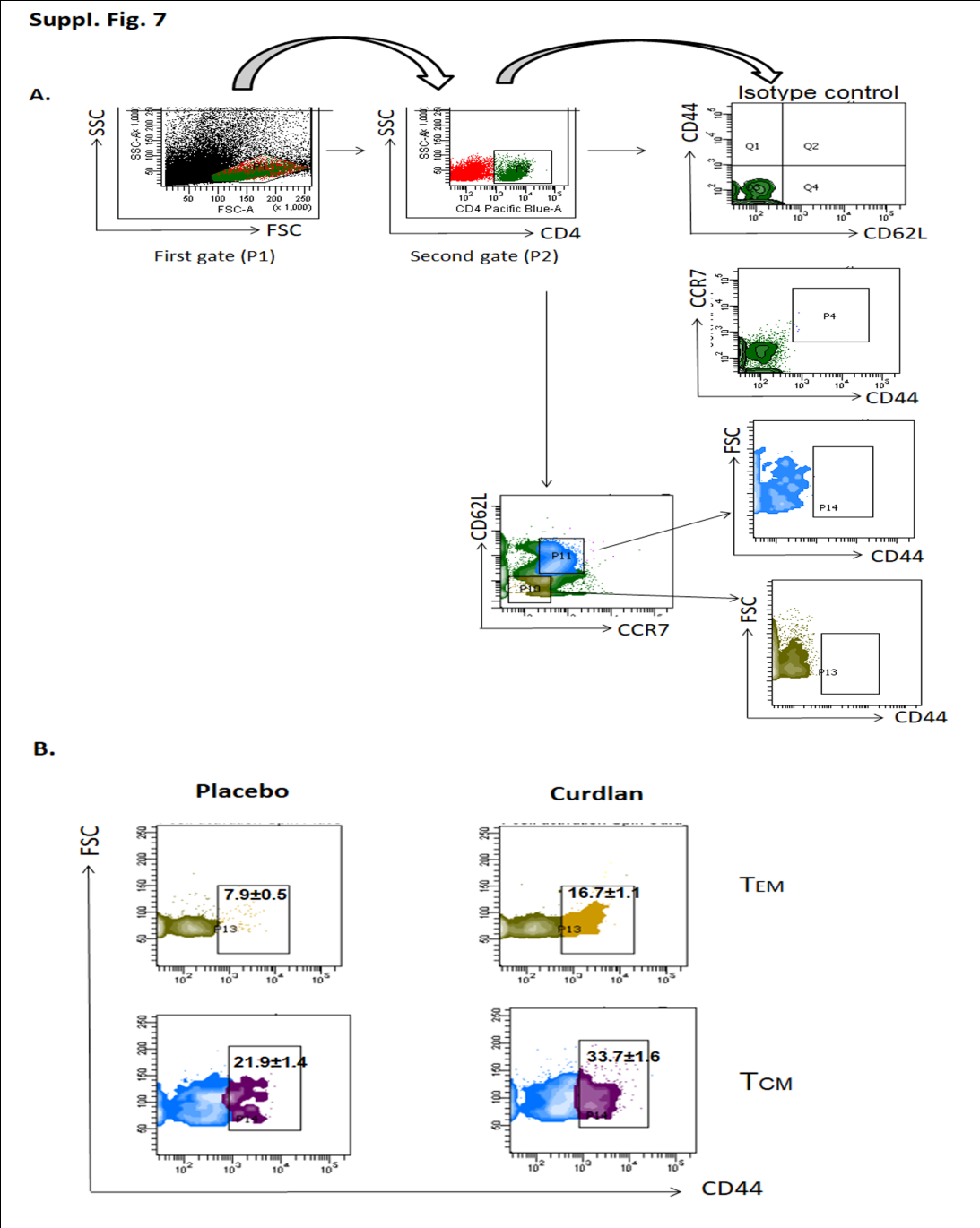

Supplement: Supplementary file 1 [file Data_Sheet_1.doc]
